# Structural basis for an exceptionally strong preference for asparagine residue at the S2 subsite of *Stenotrophomonas maltophilia* dipeptidyl peptidase 7

**DOI:** 10.1038/s41598-021-86965-x

**Published:** 2021-04-12

**Authors:** Akihiro Nakamura, Yoshiyuki Suzuki, Yasumitsu Sakamoto, Saori Roppongi, Chisato Kushibiki, Natsuri Yonezawa, Masato Takahashi, Yosuke Shida, Hiroaki Gouda, Takamasa Nonaka, Nobutada Tanaka, Wataru Ogasawara

**Affiliations:** 1 Department of Science of Technology Innovation, Nagaoka University of Technology, 1603-1 Kamitomioka, Nagaoka, Niigata 940-2188 Japan; 2 National Institute of Technology (KOSEN), Nagaoka College, 888 Nishikatakai, Nagaoka, Niigata 940-8532 Japan; 3 School of Pharmacy, Iwate Medical University, 1-1-1 Idaidori, Yahaba, Iwate 028-3694 Japan; 4 School of Medicine, Iwate Medical University, 1-1-1 Idaidori, Yahaba, Iwate 028-3694 Japan; 5 School of Pharmacy, Showa University, 1-5-8 Hatanodai, Shinagawa-ku, Tokyo, 142-8555 Japan; 6 Department of Bioengineering, Nagaoka University of Technology, 1603-1 Kamitomioka, Nagaoka, Niigata 940-2188 Japan; 7 School of Pharmacy, Kitasato University, 5-9-1 Shirokane, Minato-ku, Tokyo, 108-8641 Japan

**Keywords:** Structural biology, Enzyme mechanisms, Proteases

## Abstract

The emergence of drug-resistant bacteria has become a major problem worldwide. Bacterial dipeptidyl peptidases 7 and 11 (DPP7s and DPP11s), belonging to the family-S46 peptidases, are important enzymes for bacterial growth and are not present in mammals. Therefore, specific inhibitors for these peptidases are promising as potential antibiotics. While the molecular mechanisms underlining strict specificity at the S1 subsite of S46 peptidases have been well studied, those of relatively broad preference at the S2 subsite of these peptidases are unknown. In this study, we performed structural and biochemical analyses on DPP7 from *Stenotrophomonas maltophilia* (SmDPP7). SmDPP7 showed preference for the accommodation of hydrophobic amino acids at the S2 subsite in general, but as an exception, also for asparagine, a hydrophilic amino acid. Structural analyses of SmDPP7 revealed that this exceptional preference to asparagine is caused by a hydrogen bonding network at the bottom of the S2 subsite. The residues in the S2 subsite are well conserved among S46 peptidases as compared with those in the S1 subsite. We expect that our findings will contribute toward the development of a universal inhibitor of S46 peptidases.

## Introduction

Antimicrobial resistance is a worldwide problem^1^. As bacteria are able to circumvent the effects of all the currently available antibiotics, there is an urgent need for the development of new antibiotics with novel modes of action. However, the development of new antimicrobial agents is declining worldwide. Among bacterial pathogens, sugar non-fermenting Gram-negative bacteria (NFGNB) are the main focus of the antimicrobial resistance epidemic^2^. *Stenotrophomonas maltophilia* (formerly named *Pseudomonas maltophilia* or *Xanthomonas maltophilia*)^3^ is a NFGNB and is a multiple-drug resistant bacterium involved in opportunistic infections in immunocompromised patients^4–6^. This bacterium exhibits antimicrobial resistance against available carbapenem class of broad-spectrum antibiotics as it possesses multidrug-resistance pumps^7,8^, plasmids harbouring antibiotic resistance genes, and various gene transfer mechanisms involved in the acquisition of antimicrobial resistance.

The World Health Organisation (WHO) recently classified *S. maltophilia* as one of the leading multidrug-resistant organisms (MDROs) in hospital settings^9^. A multitude of factors have been associated with acquiring *S. maltophilia* infections. These include, underlying malignancy (especially hematologic malignancy), organ transplantation, human immunodeficiency virus (HIV) infection, cystic fibrosis, prolonged hospitalisation, intensive care unit (ICU) admission, mechanical ventilation, indwelling catheters (vascular, urinary, biliary), and corticosteroid or immunosuppressive therapy, (treatments are concurrently given along with antibiotics administration)^10^. Considering the wide range of these risk factors, novel antimicrobial strategies are needed to combat *S. maltophilia* infections, and these antimicrobials must be highly specific to a certain group of bacteria to avoid the emergence of antimicrobial-resistant bacteria.

Family S46 peptidases are serine proteases showing dipeptidyl peptidase activity and are widely distributed in Bacteroidetes and Proteobacteria, but are not found in mammals^11,12^. Since S46 peptidases are important enzymes for the growth of bacteria^13,14^, these peptidases are anticipated as a novel molecular target of antibiotics^15^. However, a clear difference in the specificity at the P1 position of the substrate between the two types of S46 peptidases has been an obstacle when designing a universal inhibitor. S46 peptidases can be divided into two types, dipeptidyl peptidases 7 (DPP7) and dipeptidyl peptidase 11 (DPP11) according to the specificity at the P1 position (NH_2_-P2-P1-P1′-P2′-…, where the P1-P1′ bond is the scissile bond)^16^. DPP11s exhibit a strict specificity for acidic residues (Asp/Glu) at the P1 position, whereas DPP7s exhibit a broad specificity for both aliphatic and aromatic residues at the P1 position. Crystal structure analyses have unravelled the substrate recognition mechanisms of S46 peptidase at the P1 position. S46 peptidases have two subunits forming a homodimer, with each subunit consisting of approximately 770 amino acid residues. The first three-dimensional structure of an S46 peptidase was determined for dipeptidyl aminopeptidase BII (DAP BII), a DPP7-type enzyme, from *Pseudoxanthomonas mexicana* WO24^17^. The study revealed that a protomer of DAP BII contains a peptidase domain, including a double β-barrel fold that is characteristic of the chymotrypsin superfamily, as well as an unusual α-helical domain that regulates the exopeptidase activity of DAP BII. Subsequently, crystal structures of dipeptidyl peptidase 11 from *Porphyromonas gingivalis* (PgDPP11) were identified^18,19^. Crystal structure analyses and biochemical studies of PgDPP11^16,20^ have revealed that Arg673 in PgDPP11 is responsible for the strict Asp/Glu specificity of PgDPP11 at the P1 position of the substrate peptide. The P1 preference of S46 peptidases could be inferred according to the amino acid at position 673 of PgDPP11^16^. DPP7s have glycine, and DPP11s have arginine or serine at position 673 of PgDPP11. As DPP7s have glycine at position 673, the S1 subsite of DPP7s can form a pocket deep enough to accommodate bulky hydrophobic residues. On the other hand, DPP11s exhibit specificity for Asp/Glu by the electrostatic interaction between the side chain of Arg673 and the carboxy group of the P1-side chain of Asp/Glu^18^. Thus, the overall structure, the molecular basis of the exopeptidase activity, the catalytic mechanism, and the strict P1-residue recognition mechanisms of S46 peptidases have been unravelled by the crystal structure analyses of DAP BII and PgDPP11^17–19^. In addition, with regards to the recognition of the prime side (P1′-P2′-…) of the substrate peptide, it has been reported that specific interactions between the prime side subsites (S1′-S2′-…) and the side chain of the substrate (P1′-P2′-…) were not observed in the crystal structure of DAP BII complexed with an octapeptide^17^. Because antibiotics targeting S46 peptidases need to inhibit both DPP7 and DPP11, this specificity difference between DPP7 and DPP11 at the P1 position can be a potential obstacle when designing a universal inhibitor of S46 peptidases. However, little is known about the mechanism of P2-residue recognition.

Biochemical studies of DPP7 from *P. gingivalis* (PgDPP7) and PgDPP11 suggested that the side chain of Phe664 in PgDPP7 and of Phe671 in PgDPP11 are involved in the recognition of the hydrophobic P2 residue of the substrate peptide. This hydrophobic specificity at the P2 position is conserved between DPP7 and DPP11^21^. The conservation of specificity at the P2 position has provided access to design the universal inhibitor of S46 peptidases, however, determinants for the P2-residue specificity/preference of S46 peptidases at the atomic level remain to be fully elucidated. It should be noted that in mammals, a peptidase designated DPP7 is one of the two members of the S28 peptidase family^22,23^ and is distinct from bacterial DPP7s that belong to the S46 peptidase family. The S28 peptidases contain two major domains: a peptidase domain, including an α/β-hydrolase fold, and an α-helical bundle. Therefore, understanding the substrate recognition mechanism at the S2 subsite could provide more insight for the development of a universal inhibitor of S46 peptidases.

In this study, we evaluated the P2-residue preference of DPP7 from *S. maltophilia* (SmDPP7) and determined the crystal structures of SmDPP7 in complexes with four kinds of dipeptides (Val-Tyr, Phe-Tyr, Tyr-Tyr, and Asn-Tyr) at resolutions of 2.03 to 1.86 Å. Biochemical studies showed that SmDPP7 prefers hydrophobic amino acids at the S2 subsite in general, but as an exception, also for asparagine. Crystal structure analysis and isothermal titration calorimetry (ITC) analysis of the Asn-Tyr bindings with SmDPP7 revealed that the exceptionally strong preference for asparagine residue is facilitated by a water-mediated hydrogen bond network in the S2 subsite. An extensive amino acid sequence comparison also revealed that residues in the S2 subsite of S46 peptidases are better conserved than those in the S1 subsite. Indeed, competitive inhibition assay using dipeptides against other S46 peptidases from pathogenic bacteria showed a conserved preference for hydrophobic residues and asparagine at the S2 subsite. These observations provide novel insights into the design of a universal inhibitor of S46 peptidases.

## Results

### P2 position preferences of SmDPP7

**Figure 1:**
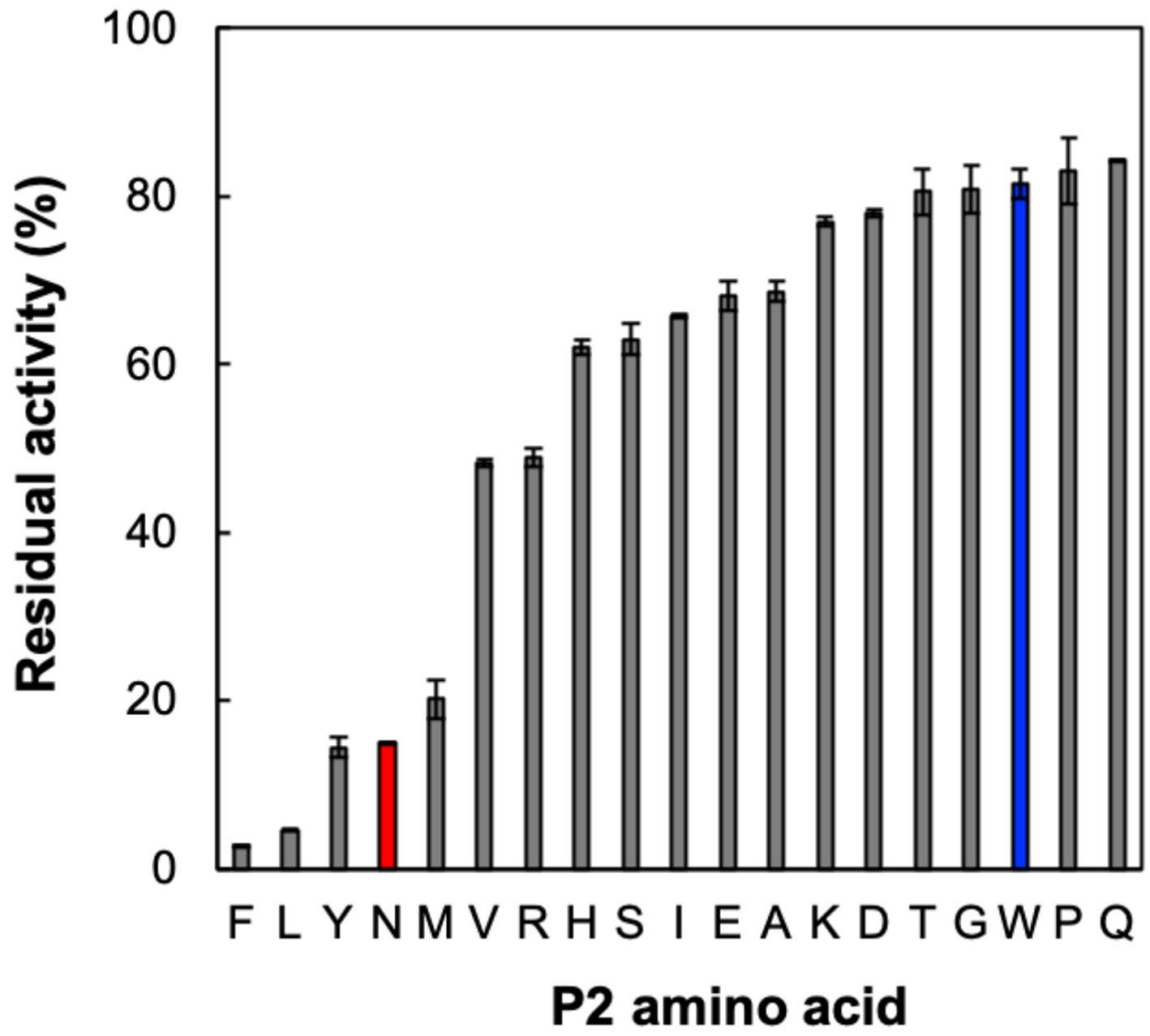
Effect of Xaa-Tyr dipeptides on the hydrolytic activity of SmDPP7 towards synthetic substrate Tyr-Tyr-MCA. Inhibition assay was performed as described in “Materials and methods”. Residual activity was measured under conditions where 200 µM Xaa-Tyr dipeptide and 200 µM Tyr-Tyr-MCA, synthetic substrate were added into the reaction solution. The outlier dipeptides Asn-Tyr and Trp-Tyr are coloured in red and blue, respectively (see text and Fig. 2a). The standard deviations were obtained from three independent experiments.

**Figure 2:**
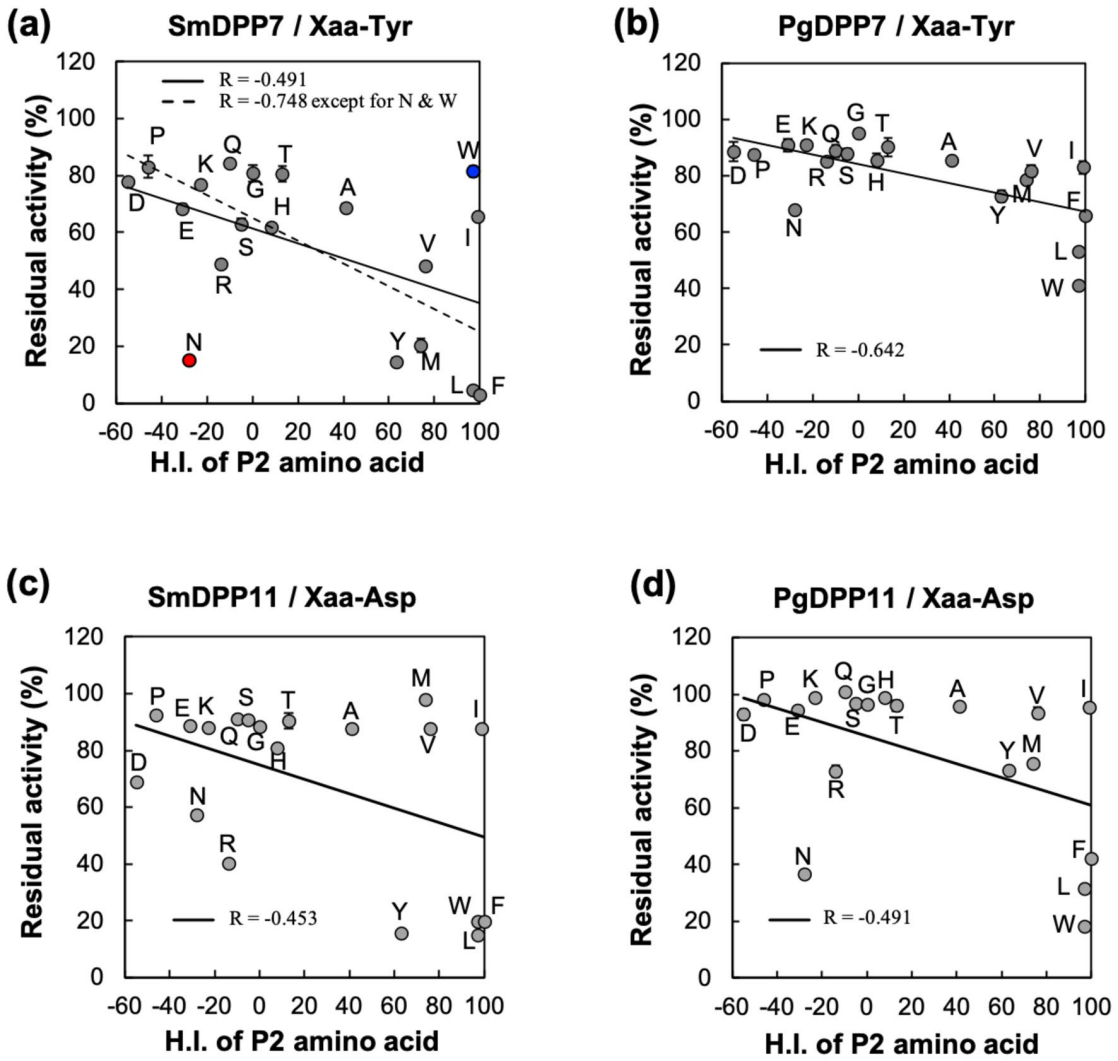
Correlation diagrams between the hydrophobicity index (H.I.) of P2-amino acid and residual DPP activities of S46 peptidases. (**a**) SmDPP7. The outlier dipeptides Asn-Tyr and Trp-Tyr are coloured in red and blue, respectively (see text). (**b**) PgDPP7. Tyr-Tyr-MCA hydrolysis of PgDPP7 was inhibited by Xaa-Tyr dipeptide. Residual activity was measured under conditions where 200 µM dipeptides and 200 µM Tyr-Tyr-MCA were added into the reaction solution. (**c**) DPP11 from *S. maltophilia* (SmDPP11). (**d**) PgDPP11. For DPP11s, Leu-Asp-MCA hydrolysis was inhibited by Xaa-Asp dipeptide. Residual activity was measured under conditions where 100 µM dipeptides and 100 µM Leu-Asp-MCA were added into the reaction solution. The hydrophobicity indices of the amino acids were adopted from Sereda et al., 1994 and Mohera et al. 1995^25,26^. R is the correlation coefficient. The standard deviations were obtained from three independent experiments.

First, we evaluated the P1 preferences of SmDPP7, SmDPP11, PgDPP7, and PgDPP11 by using synthetic substrates dipeptidyl-*p*-nitroanilide (Figure S1). SmDPP7 showed clear preferences for P1-hydrophobic amino acids and an ability to degrade a dipeptidyl substrate with basic amino acid at the P1 position. This result was in agreement with previous reports, in which PmDAP BII and PgDPP7 showed a preference against P1-hydrophobic amino acids and an ability to degrade dipeptidyl substrates consisting of basic amino acids at the P1 position^11,16,21,24^. In order to quantitatively estimate the preference at the P2 position of the substrate, inhibitory effects of the dipeptides Xaa-Tyr against the hydrolytic activity of wild-type SmDPP7 were evaluated on a synthetic substrate, l-tyrosyl-l-tyrosyl-4-methylcoumaryl-7-amide (Tyr-Tyr-MCA). Here, Xaa indicates any amino acid except for Cys, because Cys-Tyr was not included in the commercially available dipeptide library (AnaSpec, Fremont, CA, US) and the synthetic service (Eurofins Genomics, Japan) that we used. The hydrolytic activity of SmDPP7 was markedly inhibited by Phe-Tyr, Leu-Tyr, Tyr-Tyr, Asn-Tyr, and Met-Tyr, the top five of the 19 dipeptides shown in descending order (Fig. 1). The inhibitory constants (*K*_i_) of Phe-Tyr, Leu-Tyr, Tyr-Tyr, and Met-Tyr were 1.27, 2.39, 7.66 and 11.2 µM, respectively, and these were clearly aligned with the hydrophobicity indexes (H.I.) of P2 (N-terminal) amino acids^25,26^ (Table 1). The correlation coefficient between the residual activity of SmDPP7 and H.I. of the P2 amino acid of dipeptides was –0.748 when the outlier dipeptides Asn-Tyr and Trp-Tyr (red and blue, respectively, in Fig. 2a) were excluded, whereas the value was –0.491 when all the dipeptides were considered. The negative correlations between the residual activity and the H.I. of the P2 amino acid of dipeptides indicated that SmDPP7 prefers the hydrophobic amino acids at the P2 position of the substrate. Interestingly, Asn-Tyr (H.I. of Asn: –28) showed an exceptional inhibitory effect with *K*_i_ value of 7.80 µM against SmDPP7. In order to evaluate the characteristics of the Asn-Tyr as N-terminal part of the substrate of SmDPP7, the kinetic parameters of SmDPP7 for Asn-Tyr-MCA were determined (Table 2). The specificity constant (*k*_*cat*_/*K*_m_) for Asn-Tyr-MCA of wild-type SmDPP7 was 71.5 s^-1^ mM^-1^, which was 1.5 times higher than that for Tyr-Tyr-MCA. This result indicated that Asn was accommodated into the S2 subsite of SmDPP7 not only as the N-terminal residue of the product dipeptide (Asn-Tyr) but also as the P2 residue of substrate peptide. We further examined the binding of amino acids at the S2 subsite of SmDPP7 by structural and site-directed mutagenesis studies, as described below.

**Table 1:** Inhibition constants (*K*_i_) of Xaa-Tyr/Xaa-Asp dipeptides against the hydrolytic activities on synthetic substrates of S46 peptidases.

P2 amino acid (Xaa)	H.I. of P2 aa	*K*_i_ (µM)			
		SmDPP7	PgDPP7	SmDPP11	PgDPP11
Asp	− 55	–	–	–	–
Pro	− 46*	–	–	–	–
Glu	− 31	–	–	–	–
Asn	− 28	7.80 ± 0.42	210 ± 18	61.9 ± 6.6	4.06 ± 0.16
Lys	− 23	–	–	–	–
Arg	− 14	–	–	23.2 ± 1.4	–
Gln	− 10	–	–	–	–
Ser	− 5	–	–	–	–
Gly	0	–	–	–	–
His	8	–	–	–	–
Thr	13	–	–	–	–
Ala	41	–	–	–	–
Tyr	63	7.66 ± 1.27	–	5.66 ± 0.23	–
Met	74	11.2 ± 0.4	–	–	–
Val	76	55.2 ± 0.5	–	–	–
Trp	97	–	30.6 ± 0.9	6.27 ± 0.18	1.36 ± 0.06
Leu	97	2.39 ± 0.08	61.0 ± 1.2	5.68 ± 0.07	3.35 ± 0.23
Ile	99	–	–	–	–
Phe	100	1.27 ± 0.06	150 ± 2	7.01 ± 0.27	5.11 ± 0.06

Xaa-Tyr and Xaa-Asp were used as competitive inhibitors for DPP7s and DPP11s, respectively, and Tyr-Tyr-MCA and Leu-Asp-MCA were used as substrates for DPP7s and DPP11s, respectively. The hydrophobicity indexes (H.I.) of the amino acids were adopted from Sereda et al. and Mohera et al.^25,26^.

*The hydrophobicity index of proline is normalized from Sereda et al., under the condition at pH 2.0. “–” means not determined due to low inhibitory activity. Standard deviations were obtained from three independent experiments.

**Table 2:** Kinetic parameters of wild-type and mutant SmDPP7s toward each synthetic substrate.

Variation	Substrate	*K*_m_ (µM)	*k*_*cat*_ (s^−1^)	*k*_*cat*_/*K*_m_ (s^−1^ mM^−1^)
Wild type	Gly-Tyr-MCA	183 ± 4	0.702 ± 0.022	3.85 ± 0.03
	Tyr-Tyr-MCA	81.7 ± 3.4	3.83 ± 0.09	46.9 ± 1.2
	Asn-Tyr-MCA	51.3 ± 1.6	3.67 ± 0.12	71.5 ± 0.3
K206A	Gly-Tyr-MCA	201 ± 10	0.751 ± 0.015	3.73 ± 0.13
	Tyr-Tyr-MCA	55.8 ± 1.5	3.52 ± 0.04	63.2 ± 1.5
	Asn-Tyr-MCA	54.1 ± 2.1	3.47 ± 0.02	64.4 ± 2.6
R218A	Gly-Tyr-MCA	–	–	–
	Tyr-Tyr-MCA	–	–	–
	Asn-Tyr-MCA	–	–	–
R218Q	Gly-Tyr-MCA	717 ± 63	0.0365 ± 0.0018	0.0510 ± 0.0020
	Tyr-Tyr-MCA	275 ± 11	0.0403 ± 0.0004	0.146 ± 0.005
	Asn-Tyr-MCA	390 ± 14	0.198 ± 0.006	0.509 ± 0.020
R218K	Gly-Tyr-MCA	586 ± 40	0.0238 ± 0.0011	0.0407 ± 0.0017
	Tyr-Tyr-MCA	251 ± 6	0.240 ± 0.003	0.955 ± 0.012
	Asn-Tyr-MCA	277 ± 6	0.183 ± 0.008	0.660 ± 0.017
T220A	Gly-Tyr-MCA	215 ± 10	0.454 ± 0.010	2.12 ± 0.06
	Tyr-Tyr-MCA	46.4 ± 2.4	1.82 ± 0.07	39.2 ± 0.7
	Asn-Tyr-MCA	66.6 ± 1.5	2.60 ± 0.04	39.0 ± 0.3
F671A	Gly-Tyr-MCA	688 ± 38	0.191 ± 0.009	0.278 ± 0.003
	Tyr-Tyr-MCA	463 ± 88	1.54 ± 0.18	3.36 ± 0.23
	Asn-Tyr-MCA	17.4 ± 1.4	0.230 ± 0.012	13.7 ± 0.4

Kinetic parameters were determined by fitting the experimental data to the Michaelis–Menten equation. “–” means not determined due to low activity. Standard deviations were obtained from three independent experiments.

### The overall structure of SmDPP7

**Figure 3:**
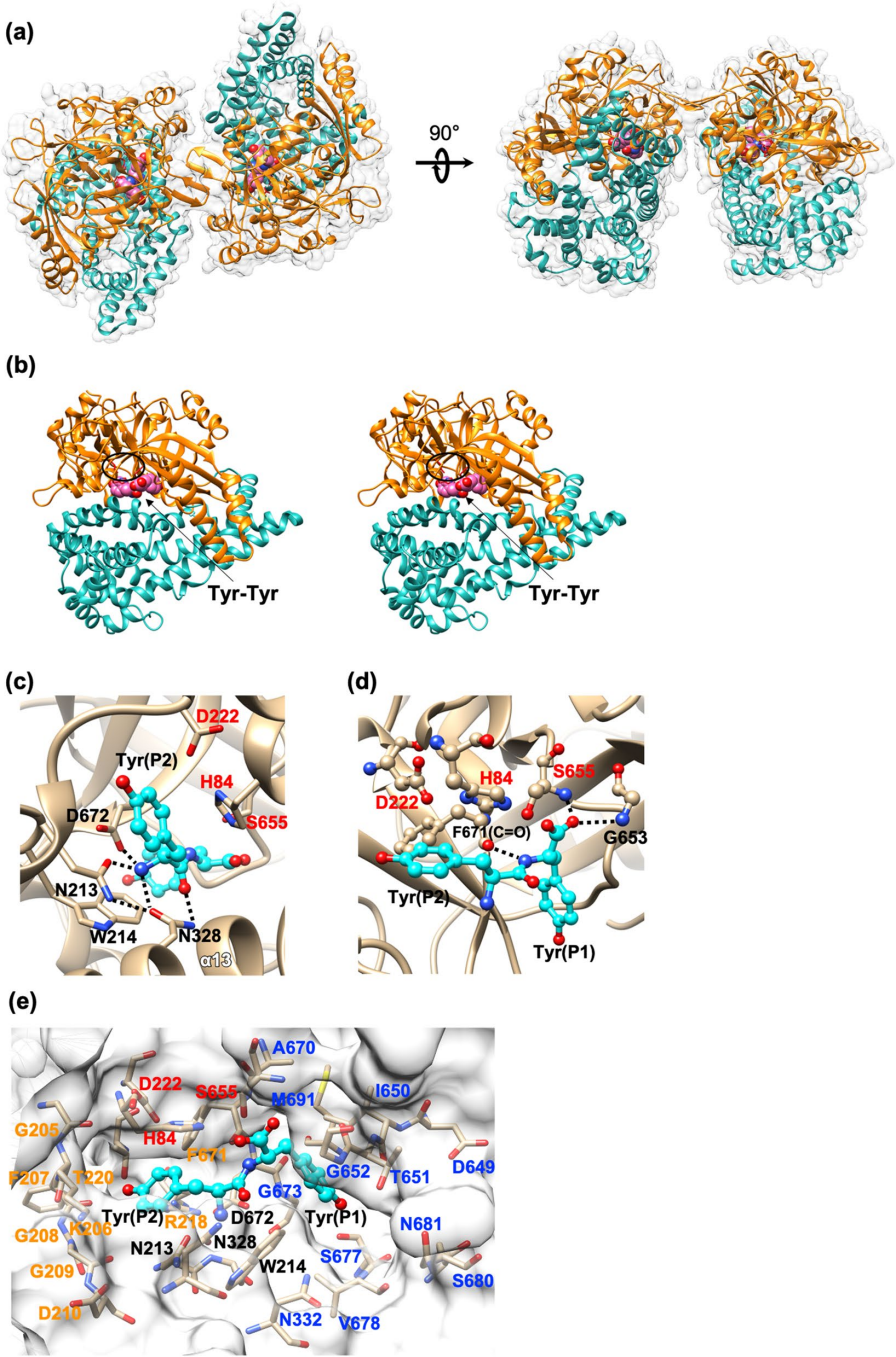
Three-dimensional structure of SmDPP7 complexed with Tyr-Tyr dipeptide. (**a**) Three-dimensional structure of dimeric SmDPP7 complexed with Tyr-Tyr dipeptide (PDB, 7DKC). Orange and sea green represent catalytic domain (residues 23–274 and 572–720) and α-helical domain (residues 275–571), respectively. The bound Tyr-Tyr dipeptide is coloured in pink. (**b**) Wall-eye stereo view of monomeric SmDPP7. The catalytic triad “Asp222-His84-Ser655” (red) is marked by an ellipsoid. The other colour codes are the same as in (**a**). (**c**) N-terminus recognition. Catalytic residues and N-terminal recognition residues are represented in red and black, respectively. (**d**) Oxyanion hole. Catalytic residues are represented in red. (**e**) S1 and S2 subsite. S1 subsite residues and S2 subsite residues are represented in blue and orange, respectively. The other colour codes are the same as in (**c**). These figures were produced using the program UCSF Chimera version 1.14^47^.

Crystal structures of SmDPP7 in complexes with dipeptides Val-Tyr, Phe-Tyr, Tyr-Tyr, and Asn-Tyr were determined at resolutions of 2.03–1.86 Å (Tables S1 and S2). Representative electron density maps of the bound dipeptides are shown in Figure S2. The SmDPP7 enzyme forms a homodimer, with each subunit consisting of 697 residues (Ala23-Ala719) and a molecular weight of approximately 155 kDa (Fig. 3). A dimer of SmDPP7 is situated in the asymmetric unit (Fig. 3a). Two protomers of SmDPP7 are related by a non-crystallographic two-fold axis and form a dimer. Dimerisations have also been observed in the crystal structures of other S46 peptidases, PmDAP BII and PgDPP11^17–19^. The protruding β-hairpin dimerisation interface reported in the above S46 peptidases is also observed for SmDPP7. Each subunit contains a catalytic double β-barrel domain harbouring the Asp-His-Ser catalytic triad (Fig. 3b, top domain) and an α-helical domain that caps the active site (Fig. 3b, bottom domain). The assignment of the secondary structural elements is provided in Figure S3.

**Figure 4:**
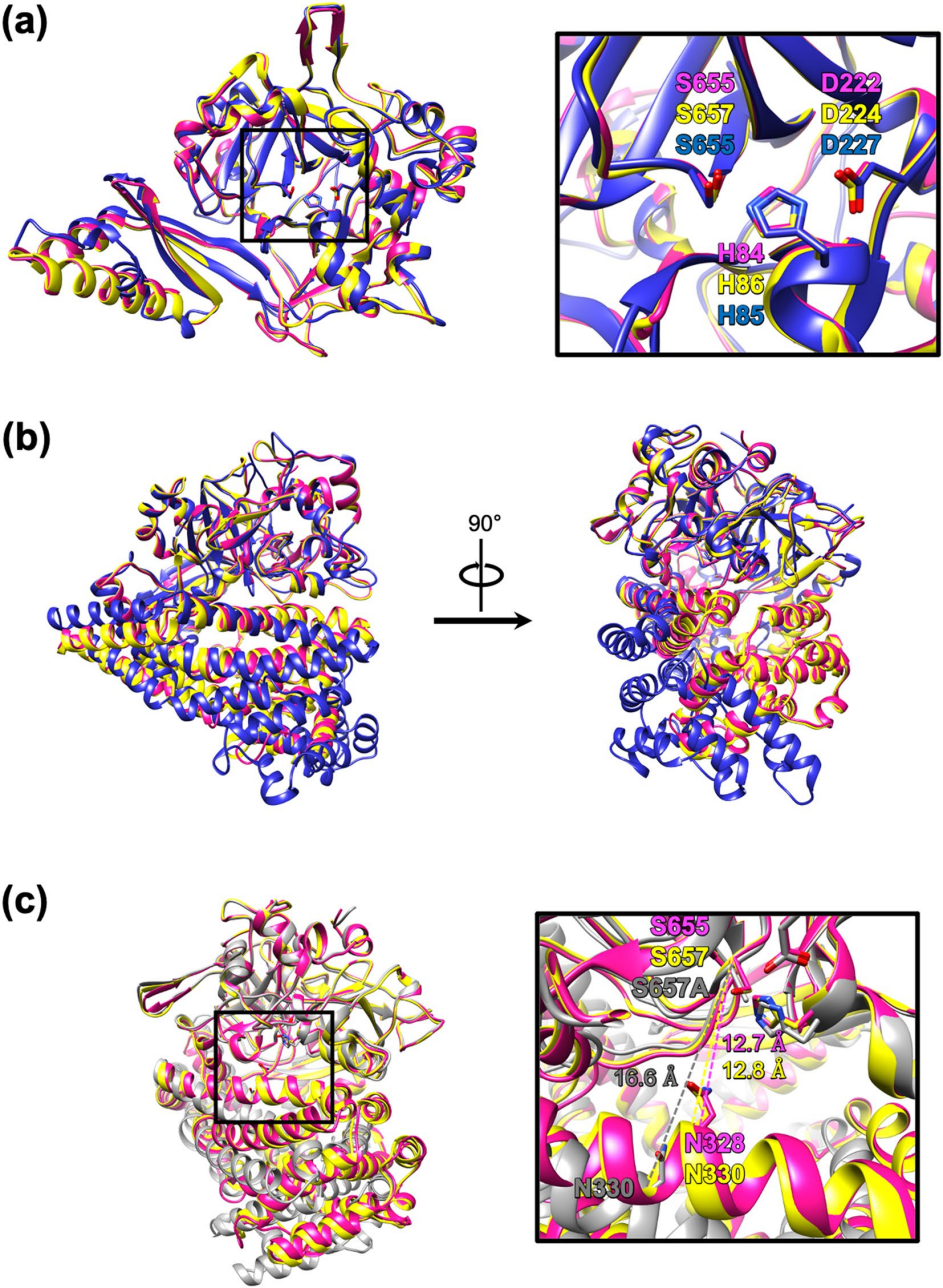
Superpositions of the protomers of SmDPP7, PmDAP BII, and PgDPP11. (**a**) Superposition of catalytic domains. Ribbon diagrams of SmDPP7 complexed with Tyr-Tyr dipeptide (PDB, 7DKC), PmDAP BII complexed with Val-Tyr dipeptide (PDB, 3WOL), and PgDPP11 complexed with citrate ion (PDB, 6JTB) are shown in magenta, yellow, and blue, respectively. Residues 23–274 and 572–720 of SmDPP7 are shown as a catalytic domain. Residues 25–276 and 574–720 of PmDAP BII and residues 22–279 and 572–720 of PgDPP11, corresponding to the catalytic domain of SmDPP7, are shown. (**b**) The superposition of the protomers of SmDPP7 (PDB, 7DKC), PmDAP BII (PDB, 3WOL), and PgDPP11 (PDB, 6JTB). The colour coding is the same as (**a**). (**c**) The superposition of the protomers of SmDPP7 and PmDAPBII. Ribbon diagrams of SmDPP7 (PDB, 7DKC), PmDAP BII (PDB, 3WOL; closed conformation), and PmDAP BII (PDB, 3WOK; open conformation) are shown in magenta, yellow, and grey, respectively. These figures were produced using the program UCSF Chimera version 1.14^47^.

The catalytic domain includes residues 23–274 and 572–720 and contains a double β-barrel structure. The β-barrel structure is a characteristic of the chymotrypsin superfamily. The catalytic domain of SmDPP7 can be superimposed on those of PmDAP BII and PgDPP11 (Fig. 4a). The serine peptidase catalytic triads, His84, Asp222 and Ser655 in SmDPP7 and His86, Asp224 and Ser657 in PmDAP BII, are almost completely superimposable, with a root mean square (rms) deviation between the two structures of 0.355 Å for 397 structurally equivalent Cα atoms that had 84.0% sequence identity for that region. Similarly, the rms deviation between the catalytic domains of SmDPP7 and PgDPP11 is 1.01 Å for 308 structurally equivalent Cα atoms that had 41.9% sequence identity for that region. Although the sequence identity of the catalytic domains between SmDPP7 and PgDPP11 is low, the catalytic triad (His, Asp, and Ser) of both enzymes can be superposed (Fig. 4a, right-side).

The α-helical domain is inserted between strands β9 and β10 of the catalytic domain and spans residues 275–571 (Figure S3). The domain consists of 15 helices packed into a helical bundle that caps the catalytic triad of the catalytic domain. No structural homologue of this domain is found in the DALI database, except for the α-helical domains of the other structurally characterised S46 peptidases, PmDAP BII and PgDPP11^17,18^. Thus, the α-helical domain is absolutely restricted to S46 peptidases. The α-helical domain of SmDPP7 can be superimposed on that of PmDAP BII and PgDPP11 (Fig. 4b). The rms deviation between the α-helical domains of SmDPP7 and PmDAP BII is 0.636 Å for 279 structurally equivalent Cα atoms across all 297 pairs, which had 68.5% sequence identity for that region. The rms deviation between the α-helical domains of SmDPP7 and PgDPP11 is 1.38 Å for 66 structurally equivalent Cα atoms across all 285 pairs, which had 19.1% sequence identity for that region. In particular, the α-helical domain of PgDPP11 exhibits different inter-domain orientation (Fig. 4b, blue) as compared with those of SmDPP7 and PmDAP BII (Fig. 4b, right). Thus, we found that the structure of the α-helical domain of SmDPP7 is similar to that of PmDAP BII but is difficult to superpose onto that of PgDPP11.

### Dipeptide complexes

The four dipeptide complexes clearly show the molecular basis for peptide recognition mechanism at the S2 and S1 sites of SmDPP7. For simplicity, the following description refers primarily to subunit A of the 1.86-Å-resolution structure of the Tyr-Tyr complex of SmDPP7. The bound dipeptide was found in the active site cleft of the catalytic domain (Fig. 3b) and was covered by the α-helical domain. SmDPP7 hydrolyses peptides from the N-terminus of oligopeptides, cleaving the dipeptide units (NH_2_-P2-P1-COOH) when the second P1 residue is a hydrophobic amino acid. To act as a dipeptidyl aminopeptidase, SmDPP7 must fix the N-terminus of the substrate peptide in position. The N-terminal amino group recognition residues of SmDPP7 are Asn213, Trp214, and Asp672 from the catalytic domain and Asn328 from the α-helical domain (Fig. 3c). For PmDAP BII, a large-scale conformational change, from open to closed, was observed upon peptide binding^17^. The distance between the Cα atoms of Asn330 belonging to the α-helical domain and catalytic Ser657 belonging to the catalytic domain of PmDAP BII observed in the peptide-free, open conformation (PDB ID: 3WOK, Fig. 4c, grey) was 16.6 Å, whereas that observed in the dipeptide-bound, closed conformation (PDB ID: 3WOL, Fig. 4c, yellow) was 12.8 Å. The corresponding distance between the Cα atoms of Asn328 and Ser655 of SmDPP7 observed in the current dipeptide-bound conformation was 12.7 Å (Fig. 4c, pink). This result suggests that the present structure of SmDPP7 corresponds to the closed conformation of PmDAP BII and that the active site cleft of SmDPP7 is closed upon peptide binding. The catalytic triad of SmDPP7 is composed of His84, Asp222, and Ser655. The hydroxy group of Ser655 is hydrogen bonded to the imidazole group of His84 (OG(Ser655)–NE2(His84): 2.9 Å). One of the oxygen atoms of the carboxy group of Asp222 forms a hydrogen-bond with His84 and completes the catalytic triad (ND1(His84)–OD2(Asp222): 2.7 Å). The oxyanion hole is formed by the backbone amide nitrogen atoms of Ser655 and Gly653, and t﻿he backbone NH group of P1 residue of the bound peptide is recognised by a hydrogen bond with the carbonyl oxygen of Phe671 (Fig. 3d).

The S1 subsite of SmDPP7 is observed adjacent to the catalytic Ser655 and the oxyanion hole (Fig. 3e). The S1 subsite consists of Asn335, Asp649, Ile650, Thr651, Gly652, Ala670, Gly673, Ser677, Val678, Ser680, Asn681, and Met691. Gly673 in SmDPP7, corresponds to Arg673 in PgDPP11, which is a crucial residue for the P1 residue specificity/preference of S46 peptidases (relatively loose preference of DPP7 and Asp/Glu specificity of DPP11)^16,18,20^, and is located in the wall of the S1 subsite. Thus, the S1 subsite of SmDPP7 is large and deep enough to accommodate any amino acid as the P1 residue of the substrate peptide. The aromatic ring of the bound Tyr(P1) has hydrophobic contacts with the side chains of Trp214 and Ile650. The hydroxy group of the bound Tyr(P1) points toward the bottom of the S1 subsite. There are four buried water molecules at the bottom of the S1 subsite and they constitute a hydrogen bond network with the main-chain and side-chain atoms in the S1 subsite. The hydroxy group of the bound Tyr(P1) is involved in the hydrogen bond network of the water molecules (not shown in Fig. 3e).

**Figure 5:**
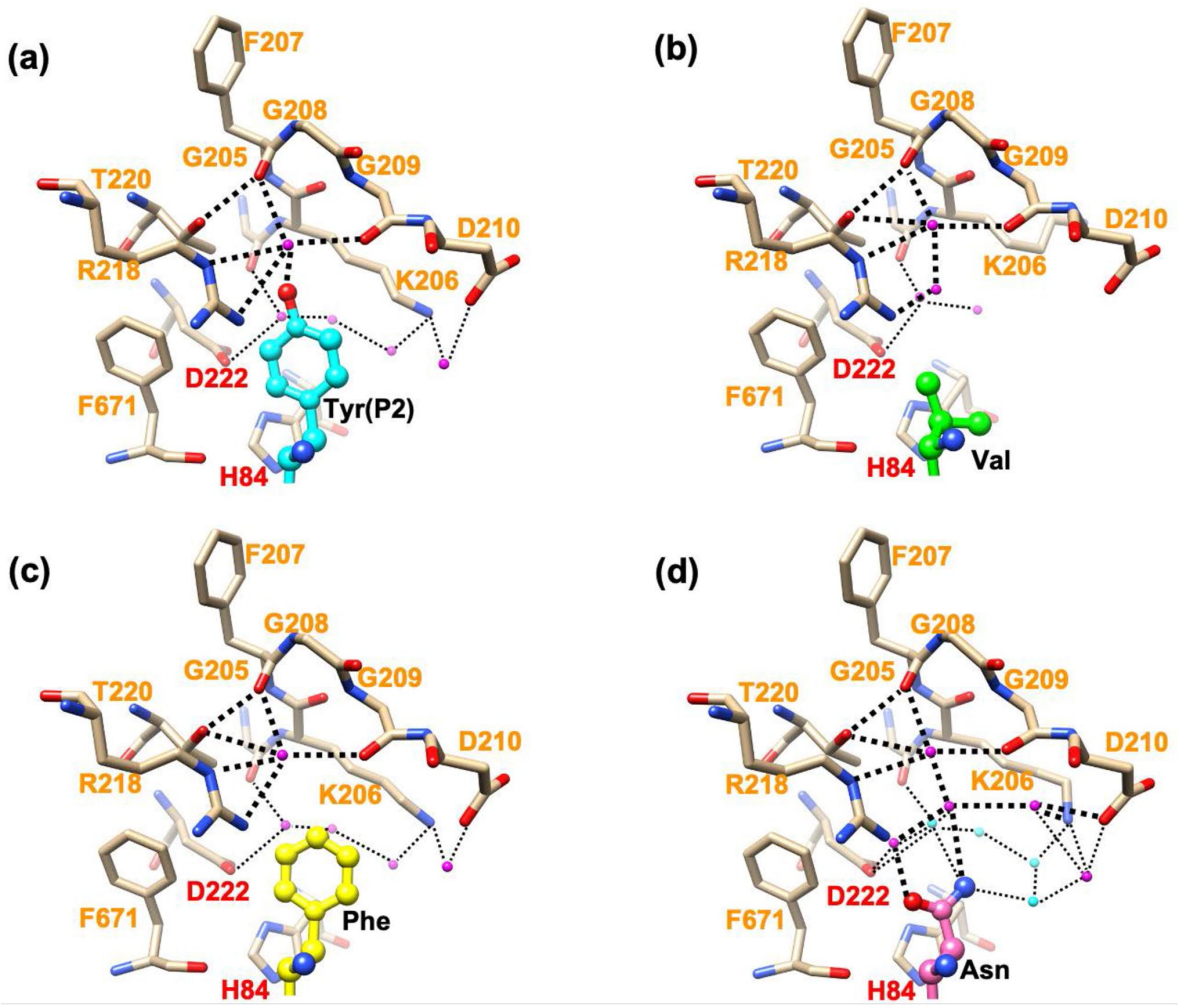
The S2 subsite of SmDPP7/dipeptide complexes. Catalytic residues and S2 subsite residues are denoted in red and orange, respectively. Magenta and cyan spheroidal denote a water molecule. (**a**) Tyr-Tyr dipeptide (cyan) complex at a 1.86-Å resolution. (**b**) Val-Tyr dipeptide (green) complex at a 2.03-Å resolution. (**c**) Phe-Tyr dipeptide (yellow) complex at a 1.91-Å resolution. (**d**) Asn-Tyr dipeptide (pink) complex at a 1.92-Å resolution. Cyan spheroids denote a water molecule associated with a pentagonal hydrogen-bond network consisting of HOH35, HOH63, HOH67, HOH1112, and the ND2 atom of P2-Asn (see Figure S4). These figures were produced using the program UCSF Chimera version 1.14^47^.

The S2 subsite of SmDPP7 composed of His84, Gly205(C=O), Lys206, Phe207(C=O), Gly208, Gly209, Asp210, Arg218, Thr220, Asp222, and Phe671 is sufficiently wide enough to accommodate a bulky side chain (Fig. 3e). The side chain of bound Tyr(P2) is accommodated in the S2 subsite and has aliphatic contacts with the side chains of His84, Lys206, and Phe671 (Fig. 5a). The hydroxy group of Tyr(P2) forms hydrogen bonds to the carbonyl oxygens of Phe207 and Gly209, and the side chain of Arg218 via a water molecule. Thus, specific interaction between the S2 subsite and the side chain of Tyr(P2) is limited, whereas the main-chain atoms of Tyr(P2) are tightly fixed by the side chains of Asn213, Trp214, Asn328, and Asp672 as described above. This is consistent with the lack of a strict specificity of SmDPP7 for the P2 residue.

For the Val-Tyr complex, some differences were observed in the intermolecular interactions around the S2 subsite as compared to those for the Tyr-Tyr complex, while intermolecular interactions between the side chain of Tyr(P1) and the residues in the S1 subsite were almost identical between the Val-Tyr and Tyr-Tyr complexes (not shown in Fig. 5b). The unique features of the Val-Tyr complex are that the bottom space of the S2 subsite is occupied by four water molecules (Fig. 5b) and the side chain of Lys206 is less ordered. In the Tyr-Tyr complex, one of the four water molecules at the bottom of the S2 subsite in the Val-Tyr complex is replaced by the hydroxy group of Tyr(P2) and the side chain of Lys206 is well ordered due to aliphatic contacts with the side chain of Tyr(P2). For the Phe-Tyr complex, intermolecular interactions around the active site are almost conserved as compared with that of the Tyr-Tyr complex. The side chain of Lys206 is well ordered and the five water molecules observed at the bottom of the S2 subsite of the Tyr-Tyr complex are also observed for the Phe-Tyr complex (Fig. 5c). For the Asn-Tyr complex, an extensive hydrogen-bond network was formed at the bottom of the S2 subsite (Fig. 5d). Interestingly, a pentagonal hydrogen-bond network consisting of HOH35, HOH63, HOH67, HOH1112, and the ND2 atom of P2-Asn was observed (Figure S4). Thus, the exceptional Asn(P2) preference of SmDPP7 could be explained by the water-mediated hydrogen-bond network at the bottom of the S2 subsite of SmDPP7.

### Site-directed mutagenesis studies on residues in the S2 subsite of SmDPP7

To test the roles of the residues located in the S2 subsite for P2 residue recognition by SmDPP7, we replaced the following residues with alanine and analysed the enzymatic activities of the mutant enzymes on synthetic substrates, Gly-Tyr-MCA, Tyr-Tyr-MCA, and Asn-Tyr-MCA (Table 2). The mutated residues and their estimated roles were: Lys206, Arg218, Thr220, and Phe671 for interaction with the P2 side chain. The Arg218 to Ala mutant (R218A) showed complete loss of activity, whereas the F671A mutant resulted in a significant loss of activity (approximately 7–20% of the *k*_*cat*_/*K*_m_ value of the wild-type enzyme). For the F671A mutant, specificity constant (*k*_*cat*_/*K*_m_) values for the Gly-Tyr-MCA, Tyr-Tyr-MCA, and Asn-Tyr-MCA substrates were significantly decreased. This result indicates that Phe671 in SmDPP7 plays an important role in the accommodation of the substrate peptide into the catalytic domain. The complete loss of the activity of R218A indicates that this residue is important for fixing the carboxy group of Asp672 in position, which is involved in the recognition of the N-terminus of the substrate peptide. The R218Q and R218K mutants retain slight dipeptidyl peptidase activities. Comparison of R218Q and R218K mutants revealed that R218K shows higher activity for all the dipeptidyl substrates examined than R218Q. The length of the Gln side chain of the R218Q mutant appears to be insufficient to fix the side chain of Asp672. The T220A mutant showed moderate loss of activity, whereas the K206A mutant retained activity comparable to that of the wild-type enzyme. For the T220A mutant, *k*_*cat*_/*K*_m_ values for all the substrates examined decreased to approximately 16–45% of the *k*_*cat*_/*K*_m_ value of the wild-type enzyme, which is attributed to the decreased *k*_*cat*_ value. It is noteworthy that *k*_*cat*_/*K*_m_ values of the K206A mutant for Tyr-Tyr-MCA and Asn-Tyr-MCA were significantly changed from that of the wild-type enzyme as compared with that for Gly-Tyr-MCA. Considering that Gly-Tyr-MCA possessed no side chain at the P2 position, the change in *k*_*cat*_/*K*_m_ values for Tyr-Tyr-MCA and Asn-Tyr-MCA of K206A suggested that the Lys206 residue is involved in an interaction with the P2 side chain.

### Thermodynamic characterisation of dipeptide bindings in SmDPP7

**Figure 6:**
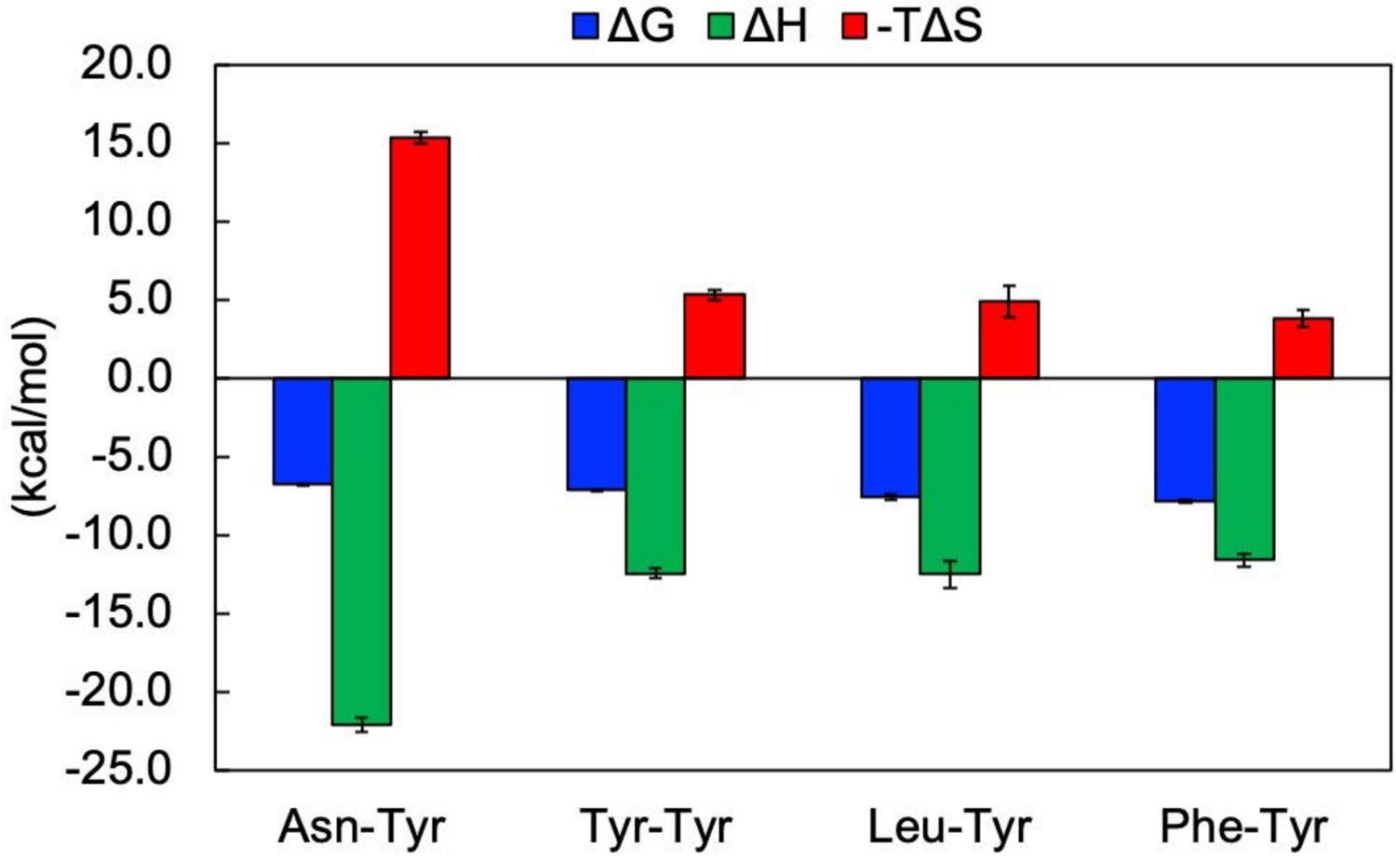
Thermodynamic parameters of dipeptide binding of SmDPP7. The calorimetric data for the respective dipeptide bindings are shown in Figure S5. The value of thermodynamic parameters is represented in Table S3. The dissociation constant (*K*_d_) and heats of binding (*ΔH*) were obtained using MICROCAL PEAQ-ITC Analysis software (Malvern, UK). Gibbs free energy (*ΔG*) and entropy energy (*ΔS*) were calculated according to the equation, *ΔG* =  − *RT* ln *K*_a_ = *RT* ln *K*_d_ (*K*_a_ = 1/ *K*_d_, association constants). Standard deviations were obtained from three independent experiments.

Crystal structure analysis of SmDPP7 complexed with Asn-Tyr indicated that a hydrogen bond network consisting of water molecules and the side chain of bound Asn contributes to the exceptional P2-Asn preference in the S2 subsite of SmDPP7, as described above. The thermodynamic parameters of dipeptide bindings in SmDPP7 were determined using isothermal titration calorimetry (ITC) (Figs. 6, S5, and Table S3). Here, we analysed bindings of five dipeptides, Asn-Tyr, Tyr-Tyr, Val-Tyr, Leu-Tyr, and Phe-Tyr. The bindings with all of the dipeptides examined in SmDPP7 were an enthalpy-driven process with an unfavourable entropic ﻿contribution. Because S46 peptidases involve conformational changes upon dipeptide binding, which reduce the degrees of freedom of the polypeptide chain (Fig. 3c)^17^, the transition from open to closed state is entropically unfavourable. For Val-Tyr, thermodynamic parameters were not determined due to low-avidity (*K*_d_ > 40 µM).

### P2 position preference of S46 peptidases

To clarify whether the P2-preferences at the S2 subsite of S46 peptidases are conserved, we analysed P2 residue preference for other S46 peptidases, PgDPP7, SmDPP11 (DPP11 from *S. maltophilia*), and PgDPP11 (Fig. 2b–d). The correlation coefficients between the residual activities of the peptidases and the H.I. of the P2 residue of dipeptides used as competitive inhibitors were – 0.642, – 0.453, and – 0.491, for PgDPP7, SmDPP11, and PgDPP11, respectively. The S46 peptidases showed a preference for hydrophobic amino acids at the P2 position of the substrate, this tendency was particularly obvious for DPP7s. The preferences of Trp(P2) for SmDPP7, and Met(P2) and Arg(P2) for SmDPP11 were out of trend and exceptional. Interestingly, Asn(P2) preference was conserved among the S46 peptidases analysed. A dipeptide harbouring Asn at the P2 position (Asn-Tyr or Asn-Asp) inhibited PgDPP7, PgDPP11, and SmDPP11 with *K*_i_ values of 210, 4.06, and 61.9 µM, respectively (Table 1). These results indicated that substrate recognition mechanisms at the S2 subsite are partly conserved between DPP7s and DPP11s.

## Discussion

In this study, we evaluated the P2 residue preference of DPP7 from *S. maltophilia* (SmDPP7), which is distinct from mammalian DPP7s that belong to the S28 peptidase family, to better understand the determinants for P2 residue specificity/preference of S46 peptidases. We solved the crystal structures of SmDPP7 in the presence of four kinds of dipeptides, Val-Tyr, Phe-Tyr, Tyr-Tyr, and Asn-Tyr. SmDPP7 showed a preference for bulky hydrophobic amino acids (except for Trp) and Asn at the P2 position of the substrate (Figs. 1, 2a, and Table 1). It should be noted that inhibition assays with dipeptides consisting of the same P1 amino acids reflect P2 preference of DPPs. The preference for bulky hydrophobic amino acids at the P2 position is reasonable considering the structure of the S2 subsite, which is sufficiently wide enough to accommodate the large side chain (Fig. 3e). Preference for hydrophobic residues at the P2 position by SmDPP7 is in agreement with previous reports^21,24^. Rouf et al*.* reported that PgDPP7 and PgDPP11 exhibited a preference for hydrophobic amino acids at the P2 position of various dipeptidyl substrates and that the Phe664 (PgDPP7 numbering) is involved in the recognition of P2 hydrophobic amino acids^21^. In this study, the F671A mutant of SmDPP7 (which corresponds to the Phe664 in PgDPP7) showed a remarkably decreased specificity constant toward dipeptidyl substrates with hydrophobic and hydrophilic amino acids at the P2 position (Table 2). This indicated that the side chain of Phe671 (numbering in SmDPP7) appears to be important for maintaining the conformation of the S2 subsite rather than for the recognition of P2 hydrophobic amino acids. However, the specificity for Asn(P2) residue by DPPs has never been reported for bacterial DPPs or mammalian DPPs^27^. To confirm whether the preference for bulky hydrophobic amino acids and Asn at the P2 position was conserved in S46 peptidases, we performed a multiple sequence alignment analysis against 4931 genes coding S46 peptidases. We observed that the residues in the S2 subsite are highly conserved among S46 peptidases as compared with those in the S1 subsite (Table S4), indicating that S46 peptidases would share a common preference at the P2 position of the substrate. Indeed, P2 preference for bulky hydrophobic amino acids and Asn are conserved among S46 peptidases examined in this study (Fig. 2). In contrast, the specificity at the P1 position of the substrate is distinct between DPP7 and DPP11 in S46 peptidases according to the lower conservation in S1 subsite residues (Figure S1 and Table S4). SmDPP7 and PgDPP7 showed a preference for P1-hydrophobic amino acids and an ability to degrade the dipeptidyl substrate with a basic amino acid at the P1 position, whereas SmDPP11 and PgDPP11 prefer the acidic amino acids at the P1 position. This indicates that the S2 subsite can be an influential target to develop the universal inhibitor of S46 peptidases.

The structures of four dipeptide complexes and results of ITC experiments clarify the dipeptide binding of SmDPP7 (Figs. 5 and 6). The binding modes of all dipeptide (reaction products) to SmDPP7 were enthalpy driven and were stabilised through the hydrogen bonds for N-terminus recognition by the side chains of Asn213, Trp214, Asp672 and Asn328 (Fig. 3c) and P1-NH group recognition by the carbonyl oxygen of Phe671 (Fig. 3d). In addition, an open-to-closed conformational change could occur for SmDPP7 when it recognises dipeptides as observed in PmDAP BII (Fig. 4c), which has 78.4% amino acid sequence identity with that of SmDPP7. Generally, recognition of a ligand molecule by strong hydrogen bonds concomitant with large-scale conformational change is mainly enthalpy driven and unfavourable in entropy^28^. In addition, the loss of conformational degrees of freedom is also entropically unfavourable for the bound ligand molecule. This is the case for SmDPP7—it recognises dipeptides by a tight hydrogen bond network (Fig. 3c,d) associated with open-to-closed conformational change. The order of dissociation constants (*K*_d_) of dipeptide (P2 a.a.; V > N > Y > L > F) was consistent with the result of competitive inhibition assay (Fig. 1 and Table S3). Among the dipeptide complexes, observations, such as strong enthalpic interactions of SmDPP7 with Asn-Tyr can be explained by an extensive hydrogen-bonding network with water molecules (Figs. 5d and 6). On the other hand, trapped water molecules in the S2 subsite are not favourable in entropy, so the Asn-Tyr binding exhibits highly unfavourable entropic contribution in ITC data (Fig. 6 and Table S3). Bindings with Tyr-Tyr, Leu-Tyr, and Phe-Tyr showed favourable enthalpic and unfavourable entropic contributions. The magnitudes of both parameters are smaller than those of Asn-Tyr binding. These thermodynamic parameters are attributed to hydrogen bonds for the dipeptide binding associated with open-to-closed conformational change. For the binding of Tyr-Tyr, Leu-Tyr, and Phe-Tyr, the ejecting water molecules from the S2 subsite as an entropy reservoir is entropically favourable. Thus totally unfavourable entropic contributions of the bindings of Tyr-Tyr, Leu-Tyr, and Phe-Tyr are explained by the fact that the favourable entropic contribution by the elimination of water molecules in the S2 subsite is smaller than the unfavourable entropic contributions by the open-to-closed conformational change and the loss of flexibility of the bound ligand molecule, which is at the same level for the four dipeptide used in this study. Indeed, ejecting water molecules were observed for Tyr-Tyr and Phe-Tyr complexes as compared with Asn-Tyr complex (Fig. 5). Binding with Leu-Tyr, which has a hydrophobic side chain at the P2 position, could also eject water molecules from the S2 subsite. In order to clarify the detail of entropic contribution, it is necessary to measure the heat capacity changes (Δ*C*_p_) and deconvolute total binding entropy (Δ*S*_tot_). Although the structure of the Tyr-Tyr complex showed hydrogen bonds to the carbonyl oxygens of Phe207 and Gly209 and the side chain of Arg218 via a water molecule (Fig. 5a), the enthalpic ﻿contribution showed little difference as compared with Phe-Tyr binding. This suggested that these hydrogen bonds do not significantly contribute to Tyr(P2) binding. To summarise, the dipeptide binding mode focused on the S2 subsite of SmDPP7, the P2-Asn is recognised by the establishment of hydrogen-bonding network at the bottom of the S2 subsite, and the bulky hydrophobic amino acids are accommodated in the S2 subsite by the hydrophobic interaction associated with the ejection of water molecules.

For DPP11 from *Porphyromonas endodontalis* (PeDPP11), the Leu-Asp (product) binding mode is energetically favourable both in enthalpy and entropy and the enthalpic contribution is dominant^19^. The binding mode includes a hydrogen-bond network involving the N-terminus and acidic residue(P1) recognition, and the ejection of solvent molecules from the inter-domain cleft concomitant with domain motion^19^. For the entropic contribution in the Leu-Asp binding of PeDPP11, the unfavourable entropic contribution concomitant with the structural change is completely offset by the favourable entropic contribution attributed to the ejecting water molecules, and the total entropic contribution is favourable. Although the substrate main chain recognition mechanism of PeDPP11 is similar to that of SmDPP7, the thermodynamic parameters observed for dipeptide binding in ITC measurements are distinct from that of SmDPP7. For SmDPP7, the unfavourable entropic contribution in dipeptide binding indicated that an unfavourable entropic contribution by conformational changes is more dominant than the favourable entropic contribution by ejecting water molecules from inter-domain cleft as an entropy reservoir. It is possible to classify DPP7 and DPP11 according to the entropic ﻿contribution in substrate binding other than the specificity at the P1 position of the substrate. The DPP7 type of S46 peptidases show an unfavourable entropic contribution that is attributed mainly to an open-to-closed conformational change, and the DPP11 type of S46 peptidases show a favourable entropic ﻿contribution that is attributed to ejecting solvent molecules from the inter-domain cleft.

As described above, a universal inhibitor of S46 peptidases is desirable to optimise for the S2 subsite. In this study, we observed that the decrease in dissociation constant values correlated with the hydrophobicity of the residue at the P2 position (Table S3). This indicates that the replacement of water molecules at the S2 subsite contributes to increasing the ﻿binding affinity to ligands associated with favourable enthalpic and entropic ﻿contributions. Enthalpically optimised inhibitors avoid bacteria with emerging resistance caused by mutation of targeted protein^29,30^. For NFGNB, mutational resistance to most antibiotics classes can arise easily^31^. Given these reports, compounds with favourable enthalpic contribution are convenient for the development of NFGNB antibiotics targeting S46 peptidases. In addition, the bacterial proton-dependent oligopeptide transporter (POT), a transport protein existing on the inner membrane, reportedly has substrate specificity against hydrophobic di- or tri-peptides^32,33^. We infer that hydrophilic compounds might be effective in inhibiting S46 peptidases that generally exist in the periplasmic space. Therefore, a compound that replaces water molecules in the S2 subsite with low log*P* values may be a valuable universal inhibitor of S46 peptidase for antibiotics of NFGNB such as *S. maltophilia* and *P. gingivalis*.

In this study, we discovered the exceptional bonding of Asn as the P2 amino acid residue to SmDPP7, a serine peptidase from the family S46, and unravelled the constituents of its S2 and S1 subsites, which could be largely responsible for the substrate recognition mechanisms of the S46 peptidases. Asn(P2) is recognised by a hydrogen-bonding network, and hydrophobic residues are accepted by hydrophobic interactions associated with ejecting water molecules from the S2 subsite. Our findings contribute toward the development of a dipeptidyl universal inhibitor of S46 peptidases, which could potentially serve as NFGNB antibiotics.

## Materials and methods

### Overexpression and purification of SmDPP7 WT and mutants

A synthetic gene coding for full-length SmDPP7 (residues 1–720), codon-optimised for expression in *E. coli*, was purchased from GenScript (Piscataway, NJ, US). The target sequence corresponding to mature SmDPP7 containing the signal peptide of DAP BIII^34^ from *Pseudoxanthomonas mexicana* WO24 was cloned into the pET22b expression plasmid (Merck, Darmstadt, Germany). The mature SmDPP7 was composed of 698 amino acids (residues 23 to 720), with a theoretical molecular weight of 75,720.95 and an isoelectric point of 8.08. Plasmids for expression of mutants, K206A, R218A, R218Q, R218K, T220A, and F671A, were obtained with overlap extension PCR using wild type expression plasmid as a template (Table S5). *E. coli* BL21 Gold (DE3) cells (Agilent Technologies, Santa Clara, CA, US) transformed with the pET22b-SmDPP7 WT and mutants expression plasmid were grown in TB media at 298 K to an OD_600_ of 0.6. Overproductions of SmDPP7 WT and mutants were induced by adding 0.1 mM Isopropyl-β-_d_-thiogalactopyranoside for 15 h at 298 K. Thereafter, the cells were harvested by centrifugation at 6000×*g*. Cells were disrupted using sonication and the cell extract was obtained by centrifuging the lysate at 20,000×*g* for 30 min. The SmDPP7 WT and mutants were purified by precipitation with 35–70% ammonium sulfate and hydrophobic column chromatography using a HiPrep 16/10 Butyl column (Cytiva, Marlborough, MA, US). The eluate was desalted using a HiPrep 26/10 desalting column (Cytiva) and finally subjected to anion-exchange column chromatography using a Mono Q 5/50 GL column (Cytiva). The fractions containing SmDPP7 WT and mutants were pooled, buffer-exchanged to 80 mM Tris/HCl pH 8.5 and concentrated to 5 mg/ml using Vivaspin 20 concentrator (Cytiva). Protein concentration was determined by Bradford assay, and a linear calibration curve was obtained using bovine gamma-globulin ranging from 0 to 0.25 mg/ml. Purity and molecular mass of purified SmDPP7 were estimated by SDS-PAGE (Figure S6).

### Overexpression and purification of PgDPP7, SmDPP11 and PgDPP11

Synthetic genes coding for full-length PgDPP7 (residues 1–712, UniProt accession number Q7MWU6) and SmDPP11 (residues 1–715, UniProt accession number B4SNQ8), codon-optimised for expression in *E. coli*, were purchased from GenScript (Piscataway, NJ, US). The target sequences corresponding to mature PgDPP7 (Asp25-Ile712) and mature SmDPP11 (Asp24-Gln715), both containing the signal peptide of DAP BIII^34^ from *P. mexicana* WO24 were amplified using PCR and cloned into the pET22b expression plasmid (Merck, Darmstadt, Germany). Overproduction and purification of PgDPP7 and SmDPP11 were performed in a way similar to those for SmDPP7 described above. Overproduction and purification of PgDPP11 has been described in the literature^18^. Purity and molecular mass of purified enzymes were estimated by SDS-PAGE (Figure S6).

### Determination of kinetic parameters toward dipeptidyl MCA

Kinetic parameters were determined by fitting the experimental data to the Michaelis–Menten equation using Excel Solver (Microsoft, WA, US) by nonlinear least-squares fitting with different concentrations of glycyl-l-tyrosyl-4-methylcoumaryl-7-amide (3.91, 7.81, 15.6, 31.3, 62.5, 125, 250, and 500 µM; Gly-Tyr-MCA; GenScript, NJ, US), l-tyrosyl-l-tyrosyl-4-methylcoumaryl-7-amide (1.56, 3.13, 6.25, 12.5, 25, 50, 100, and 200 µM; Tyr-Tyr-MCA; GenScript, NJ, US), l-asparaginyl-l-tyrosyl-4-methylcoumaryl-7-amide (1.56, 3.13, 6.25, 12.5, 25, 50, 100, and 200 µM; Asn-Tyr-MCA; GenScript, NJ, US), and l-leucinyl-l-asparaginic acid-4-methylcoumaryl-7-amide (0.781, 1.56, 3.13, 6.25, 12.5, 25, 50, and 100 µM; Leu-Asp-MCA; Peptide Institute, Osaka, Japan) as a substrate. The enzyme reaction was performed in a reaction buffer consisting of 50 mM sodium phosphate buffer pH 7.0, 5 mM EDTA, and 0.005% Tween 20 at 298 K for 20 min and standard deviations were calculated from three independent experiments. Tween 20 was added into reaction buffer to prevent proteins adsorbing to the plastic surface of the 96well plate (Figure S7). For SmDPP7 (wild-type and mutants), concentrations of the purified enzymes were 10, 2, and 2 nM for the hydrolyses of Gly-Tyr-MCA, Tyr-Tyr-MCA, and Asn-Tyr-MCA, respectively. Concentration of purified PgDPP7 used for determining kinetic parameters of Tyr-Tyr-MCA hydrolysis was 25 nM. For SmDPP11 and PgDPP11, concentrations of the purified enzymes used for determining kinetic parameters of Leu-Asp-MCA hydrolysis were 0.5 nM and 1 nM, respectively. The fluorescence intensity of the released MCA was measured with excitation at 355 nm and emission at 460 nm using an Infinite 200 PRO microplate reader (Tecan, Switzerland).

### Inhibition assay using dipeptide library

The dipeptide library was purchased from AnaSpec (Fremont, CA, US). Ile-Tyr and Arg-Tyr were synthesized by Eurofins Genomics (Tokyo, Japan). Cys-Tyr and Cys-Asp could not be purchased commercially. Different concentrations (3.13, 6.25, 12.5, 25, 50, 100, and 200 µM) of dipeptides as the inhibitor and 200 µM Tyr-Tyr-MCA (GenScript, NJ, US) as the substrate for DPP7 were added into a reaction buffer consisting of 50 mM sodium phosphate buffer pH 7.0, 5 mM EDTA and 0.005% Tween 20. For DPP11s, different concentrations (1.56, 3.13, 6.25, 12.5, 25, 50, and 100 µM) of dipeptides as the inhibitor and 100 µM Leu-Asp-MCA (Peptide Institute, Osaka, Japan) as the substrate were used. The enzyme reaction was performed at 298 K for 20 min and standard deviations were calculated from three independent experiments. Half-maximal inhibitory concentration (IC_50_) values were determined by fitting to a sigmoid curve (4-parameter logistic curve) using ImageJ software^35^ and inhibition constants (*K*_i_) were calculated using the Cheng-Prusoff Equation^36^. The fluorescence intensity of the released MCA was measured with the same method of determination of kinetics parameters toward dipeptidyl MCA.

### Crystallisation of SmDPP7

Crystals of the SmDPP7/dipeptide complexes were prepared as follows: Asn-Tyr and Val-Tyr-Pro were purchased from GenScript (NJ, US). Phe-Tyr and Tyr-Tyr-Tyr were purchased from Sigma-Aldrich (St Louis, MO, US) and Santa Cruz Biotech (Dallas, TX, SUA) respectively. The tripeptides, Val-Tyr-Pro and Tyr-Tyr-Tyr (tripeptides were used as a substrate, and the N-terminal dipeptide was observed as the bound product as described below), were dissolved in 80 mM Tris–HCl, pH 8.5 to concentrations of 30.0 mM and 20.0 mM, respectively. The 5-mg/ml SmDPP7 solution was mixed with aliquots of the respective ligand solutions at a volume ratio of 9:1 for the tripeptides Val-Tyr-Pro and Tyr-Tyr-Tyr, with final ligand concentrations of 3.0 mM and 2.0 mM, respectively. The samples were crystallised using the hanging-drop method; 1 μl of protein solution was mixed with the same volume of reservoir solution (20%(w/v) PEG8000 and 0.2 M ammonium acetate) and incubated at 293 K. The drops were suspended over 200 μl of reservoir solution in 48-well plates.

Asn-Tyr and Phe-Tyr were dissolved in purified water to concentrations of 100 mM. The 5-mg/ml SmDPP7 solution was mixed with aliquots of the respective ligand solutions at a volume ratio of 25:1 for the dipeptides Asn-Tyr and Phe-Tyr, with a final ligand concentration of 4.0 mM. The samples were crystallised using the hanging-drop method; 0.95 μl of protein solution was mixed with the same volume of reservoir solution (20%(w/v) PEG8000 and 0.2 M Calcium acetate) and incubated at 293 K. The drops were suspended over 200 μl of reservoir solution in 48-well plates.

A dipeptide complex, Tyr-Tyr complex, was obtained by co-crystallisation of SmDPP7 with a tripeptide Tyr-Tyr-Tyr, because the tripeptide Tyr-Tyr-Tyr was commercially available at a lower cost as compared with custom peptide synthesis of the dipeptide Tyr-Tyr. For the Tyr-Tyr complex, clear continuous electron density was observed for the first two residues of the tripeptide (Figure S2a), and no clear electron density was observed for the last residue. Because the SmDPP7 enzyme reaction occurred in the solution used for crystallisation, the Tyr-Tyr-Tyr (P2-P1-P1′) tripeptide acted as the substrate, and the reaction products were the N-terminal Tyr-Tyr (P2-P1) dipeptide and the C-terminal Tyr (P1′). While the N-terminal product Tyr-Tyr remained at the active site, the C-terminal product Tyr dissociated from the active site. The asymmetric unit of the Tyr-Tyr complex was composed of two independent SmDPP7 subunits; in both subunits (Fig. 3a), the hydrolysed dipeptide product (NH_2_-Tyr-Tyr-COOH), rather than a reaction intermediate, was observed (Figure S2a). Similarly, another dipeptide complex, Val-Tyr complex, was obtained by co-crystallisation of SmDPP7 with a tripeptide Val-Tyr-Pro and the hydrolysed dipeptide product (NH_2_-Val-Tyr-COOH) was observed (Figure S2b). In this case, co-crystallisation with Val-Tyr-Pro was performed to confirm the ability of imino-bond (X-Pro) hydrolysis by SmDPP7, though the dipeptide Val-Tyr was commercially available at a reasonable cost. The other dipeptide complexes (Phe-Tyr and Asn-Tyr complexes), were obtained by co-crystallisation of SmDPP7 with dipeptides Phe-Tyr and Asn Tyr, respectively (Figure S2c,d).

### X-ray data collection

For data collection under cryogenic conditions, dipeptide-complex crystals in a droplet were directly transferred to harvesting solutions [16%(w/v) PEG8000, 0.16 M ammonium acetate and 20%(v/v) glycerol] and [16%(w/v) PEG8000, 0.16 M calcium acetate and 20%(v/v) glycerol] respectively for 10 s. Crystals were mounted in nylon loops or MicroMounts (MiTeGen, Ithaca, NY, US) and flash-cooled in a cold nitrogen gas stream at 100 K immediately before data collection. Data were collected by the rotation method at 100 K using a MAR300HE CCD detector or EIGER 16 M detector with synchrotron radiation source on the beamline BL44XU at SPring-8. Laue group and unit-cell parameters were determined using the xia2/DIALS software package^37^ with XDS^38^ or MOSFLM^39^. The cell parameters and data-collection statistics are summarised in Table S1.

### Structure determination

The initial phase determination was performed for the Val-Tyr complex of SmDPP7 using the molecular replacement method. One protomer of PmDAP BII^17^ (PDB code: 3WOL), which has approximately 78% amino-acid sequence identity to SmDPP7, was used as a search model. Cross-rotation and translation functions were calculated using the MOLREP program^40^ from CCP4 suite^41^. Structure refinement was carried out with the program REFMAC5^42^, and further iterative manual model building and refinement were performed using the programs Coot^43^ and REFMAC5^42^. The stereochemistry of the model was verified using RAMPAGE^44^ and PROCHECK^45^ programs. The refined structure of the Val-Tyr complex was then used for the structural determination of the Tyr-Tyr complex by the difference Fourier method. The refined structure of the Tyr-Tyr complex was used for the initial phase determination of Asn-Tyr and Phe-Tyr complexes. Cross-rotation and translation functions were calculated using the program PHASER^46^ from the CCP4 suite^41^. After the final round of refinement, the bound dipeptide molecules were removed from the model. Then, the amplitude |*F*c| and phase angles calculated from the partial structure were used to calculate a weighted *m*|*F*o|–*D*|*F*c| omit map^42^, where ‘*m*’ is the figure of merit (approximately equal to the cosine of the phase error) and ‘*D*’ is the estimate of the coordinate error in the partial structure (Figure S2). The refinement statistics are summarised in Table S2.

### Isothermal titration calorimetry

The bindings were analysed using a MICROCAL PEAQ-ITC microcalorimeter (Malvern, UK). The binding reactions were performed in 50 mM sodium phosphate pH 7.0 and 2.5% dimethyl sulfoxide at 25 °C and were stirred at 750 rpm. A single injection of 0.4 μl and 19 times injections of 2.0 μl of the dipeptide solution were injected into 350 μl of enzyme solution (wild-type SmDPP7). The wild-type SmDPP7 concentration was 25 µM and the concentration of each dipeptide solution was 250 µM in reaction buffer. Each injection was performed for 4 s with an interval of 150 s between injections. The dissociation constant (*K*_d_) and heats of binding (*ΔH*) were obtained using MICROCAL PEAQ-ITC Analysis software (Malvern, UK). Gibbs free energy (*ΔG*) values were calculated according to *ΔG* =  − *RT* ln *K*_a_ = *RT* ln *K*_d_ (*K*_a_ = 1/ *K*_d_, association constants).

### Graphical programs

Figures 3, 4, 5, S2, and S4 were produced using the program UCSF Chimera version 1.14^47^. Figure S3 was created using Adobe Illustrator (Adobe Systems Inc., San Jose, CA, US).

## Supplementary Information

Supplementary Information

## Data Availability

Accession codes: Atomic coordinates for the reported structures have been deposited in the Protein Data Bank under accession codes 7DKB (Val-Tyr complex), 7DKC (Tyr-Tyr complex), 7DKD (Asn-Tyr complex), and 7DKE (Phe-Tyr complex). Supplementary information accompanies this paper at http://www.nature.com/scientificreports.
