# Supplementary material for: Structural basis for an exceptionally strong preference for asparagine residue at the S2 subsite of Stenotrophomonas maltophilia dipeptidyl peptidase 7
Source: Sci Rep. 2021 Apr 12;11:7929. doi: 10.1038/s41598-021-86965-x (PMC8041751; doi:10.1038/s41598-021-86965-x)
Supplement: Supplementary file 1 — Supplementary Information [file 41598_2021_86965_MOESM1_ESM.pdf]

Subject area:

ENZYME MECHANISMS, PROTEASES, STRUCTURAL BIOLOGY

*Correspondence and requests for materials should be addressed to
N.T. (tanakan@pharm.kitasato-u.ac.jp) and W.O. (owataru@vos.nagaokaut.ac.jp)

Structural basis for an exceptionally strong preference for asparagine residue at the S2 subsite of *Stenotrophomonas maltophilia* dipeptidyl peptidase 7

Akihiro Nakamura[1], Yoshiyuki Suzuki[2], Yasumitsu Sakamoto[3], Saori Roppongi[4], Chisato Kushibiki[3], Natsuri Yonezawa[3,5], Masato Takahashi[3], Yosuke Shida[6], Hiroaki Gouda[5], Takamasa Nonaka[3], Nobutada Tanaka[5,7*], and Wataru Ogasawara[1,6*].

[1]Department of Science of Technology Innovation, Nagaoka University of Technology, 1603-1 Kamitomioka, Nagaoka, Niigata 940-2188, Japan; [2]National Institute of Technology (KOSEN), Nagaoka College, 888 Nishikatakai, Nagaoka, Niigata 940-8532, Japan; [3]School of Pharmacy, Iwate Medical University, 1-1-1 Idaidori, Yahaba, Iwate 028-3694, Japan; [4]School of Medicine, Iwate Medical University, 1-1-1 Idaidori, Yahaba, Iwate 028-3694, Japan; [5]School of Pharmacy, Showa University, 1-5-8 Hatanodai, Shinagawa-ku, Tokyo 142-8555, Japan; [6]Department of Bioengineering, Nagaoka University of Technology, 1603-1 Kamitomioka, Nagaoka, Niigata 940-2188, Japan; [7]School of Pharmacy, Kitasato University, 5-9-1 Shirokane, Minato-ku, Tokyo 108-8641, Japan.

Supplementary information includes:
Figures S1-S7
Tables S1-S5

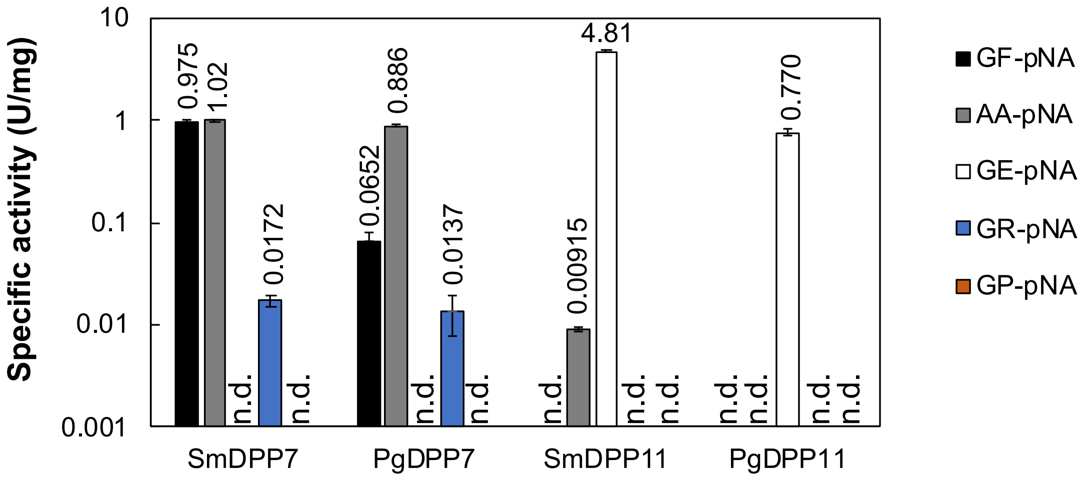

Enzyme concentrations (mg/ml) added into reaction buffer with the volume of 10 μL

		Substrates				
		Gly-Phe-pNA	Ala-Ala-pNA	Gly-Glu-pNA	Gly-Arg-pNA	Gly-Pro-pNA
Enzymes	SmDPP7	0.100	0.100	10.0	10.0	10.0
	PgDPP7	0.940	0.940	2.67	0.940	2.67
	SmDPP11	5.00	5.00	0.0142	5.00	5.00
	PgDPP11	3.70	3.70	0.161	3.70	3.70

Figure S1 | Specific activities of S46 peptidases toward synthetic substrates.

Dipeptidyl-*p*-nitroanilide (pNA) was used as a substrate at 300 μM in a 200 μL of reaction buffer consisting of 50 mM sodium phosphate buffer pH 7.0 and 5 mM EDTA. 10 μL of purified SmDPP7, PgDPP7, SmDPP11, and PgDPP11 were added into reaction buffer. Enzyme concentrations used in this assay are presented in the table above. Enzyme reaction was conducted at 310 K for 20 min. Absorbance was measured at 385 nm using GeneQuant 100 (Cytiva). One unit (U) is the amount of enzyme that released 1 μmol of pNA from dipeptidyl-pNA substrate per 1 min. "n.d." means not detected due to low activity. The standard deviations were obtained from three independent experiments.

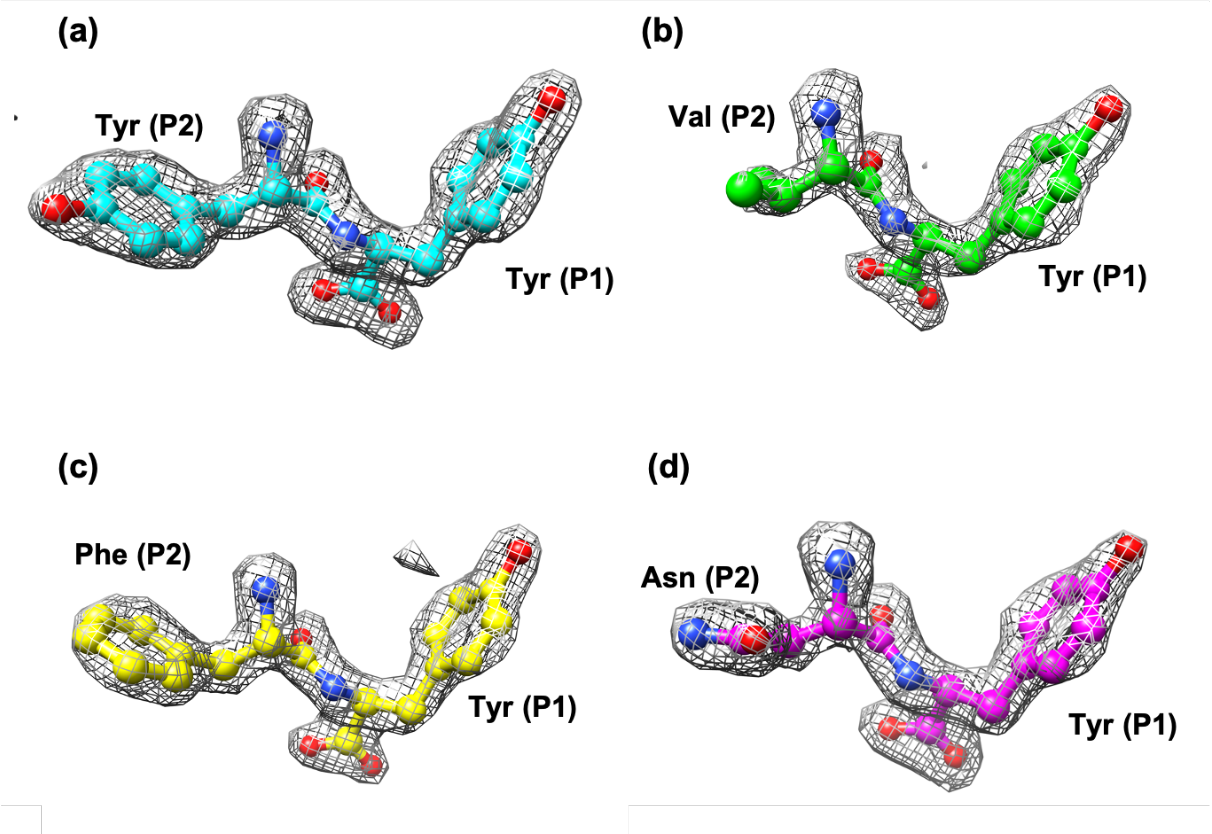

Figure S2 | Diagrams showing weighted *m*|*F*o|-*D*|*F*c| omit maps of the bound ligand molecule in the active site of SmDPP7.

The contour levels are 3.0 σ (grey). (a) Tyr-Tyr dipeptide at a 1.86-Å resolution. (b) Val-Tyr dipeptide at a 2.03-Å resolution. (c) Phe-Tyr dipeptide at a 1.91-Å resolution. (d) Asn-Tyr dipeptide at a 1.92-Å resolution. These figures were produced using the program UCSF Chimera version 1.14[1].

1. Pettersen, E. F. *et al.* UCSF Chimera—A visualization system for exploratory research and analysis. *J. Comput. Chem*. **25**, 1605–1612 (2004).

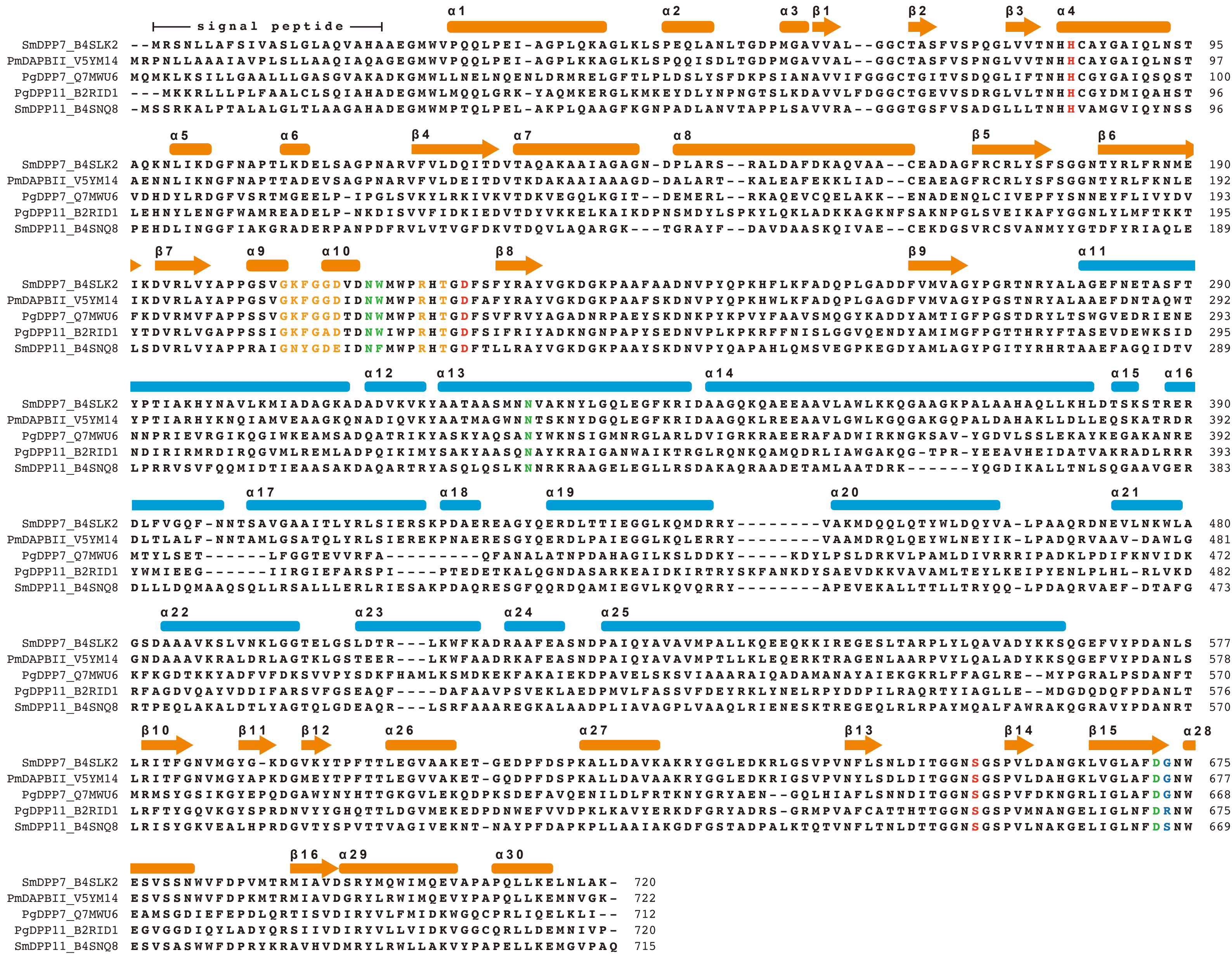

Figure S3 | Amino acid sequences of S46 peptidases mentioned in this study.

The characters following the enzyme names are the UniProt accession numbers. Secondary structural elements of SmDPP7 are shown on top. α-helices (α1-α30) and β-sheets (β1-β16) are shown in round square and arrow, respectively. Colors represent different domains: orange is the catalytic domain and blue is the helical domain. Catalytic residues, N-terminus recognition residues, and S2 subsite residues are shown in red, green, and orange, respectively. Blue represents the P1 recognition residues corresponding to position 673 amino acid of PgDPP11.

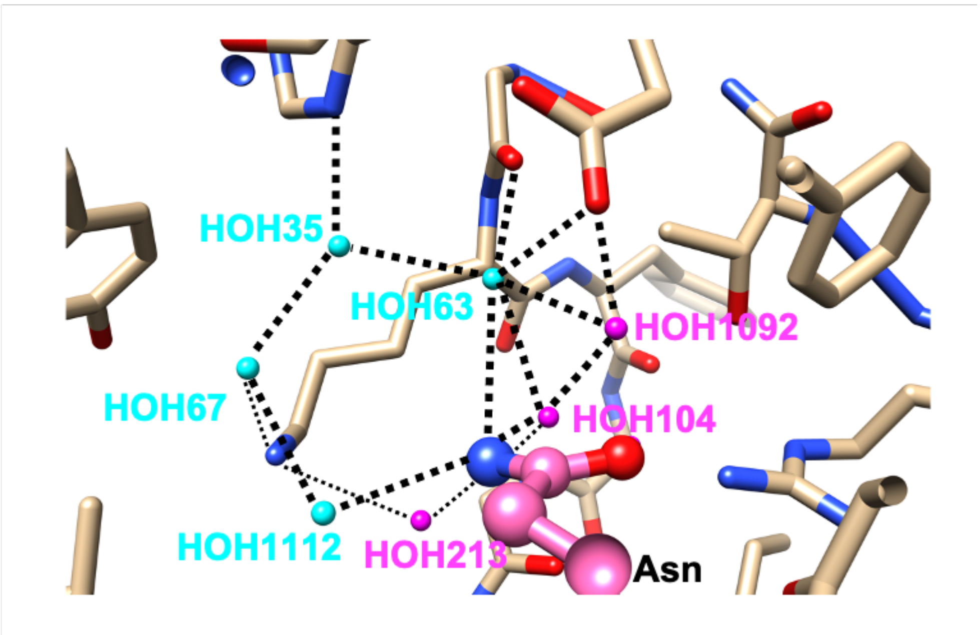

Figure S4 | A pentagonal hydrogen-bond network observed in the S2 subsite of SmDPP7/Asn-Tyr complex.

Magenta and cyan spheroidal denote water molecules. Cyan spheroids represent water molecules associated with a pentagonal hydrogen-bond network consisting of HOH35, HOH63, HOH67, HOH1112, and the ND2 atom of P2-Asn. Asn-Tyr dipeptide complex at a 1.92-Å resolution (PDB 7DKD). This figure was produced using the program UCSF Chimera version 1.14[1].

1. Pettersen, E. F. *et al.* UCSF Chimera—A visualization system for exploratory research and analysis. *J. Comput. Chem.* **25**, 1605–1612 (2004).

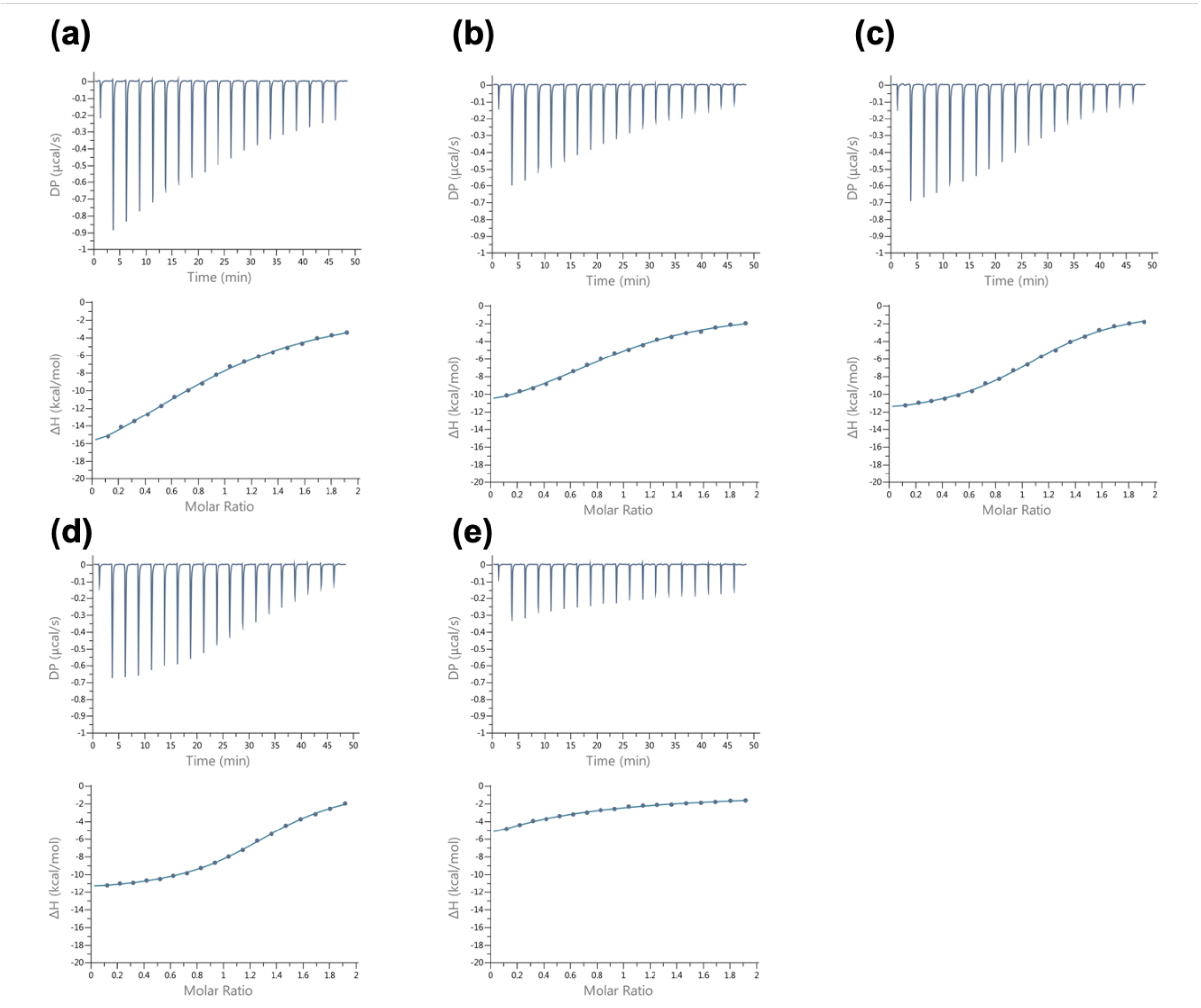

Figure S5 | Isothermal titration calorimetry (ITC) analyses of dipeptide binding to SmDPP7.

Raw data of ITC experiment depicting differential power (DP) as a function of time (top), and the graphs of fitted data using a fitting model, one-site binding model (bottom). The diagram and value of thermodynamic parameters are represented in Figure 6 and Table S3, respectively. Data from one of the three measurements are shown. (a) Asn-Tyr dipeptide. (b) Tyr-Tyr dipeptide. (c) Leu-Tyr dipeptide. (d) Phe-Tyr dipeptide. (e) Val-Tyr dipeptide.

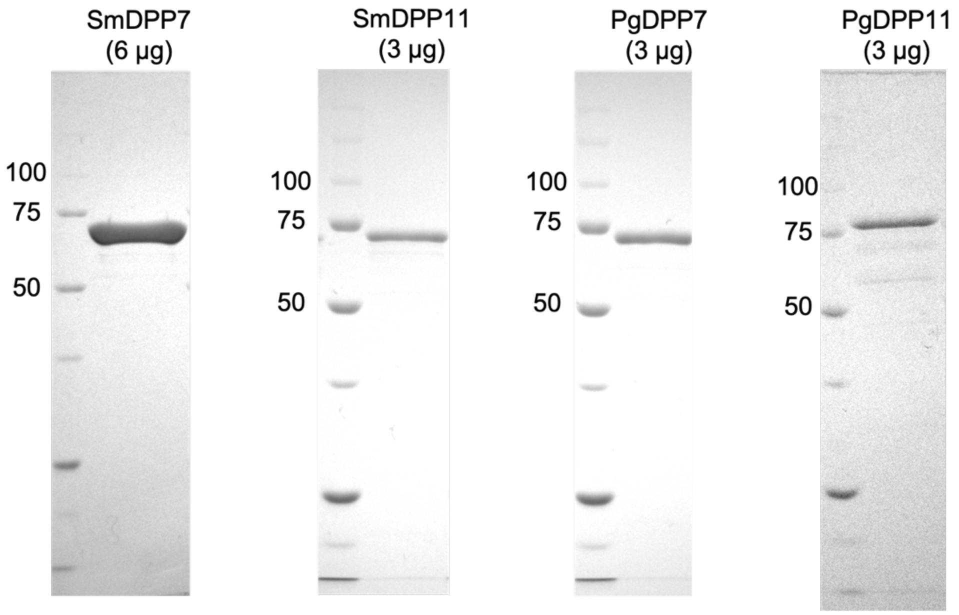

Figure S6 | Coomassie-stained 10% SDS–PAGE analysis of S46 peptidases overexpressed in this study.

Numbers in parentheses are the amount of protein loaded per lane. Molecular-weight markers of different masses (kDa) are labelled.

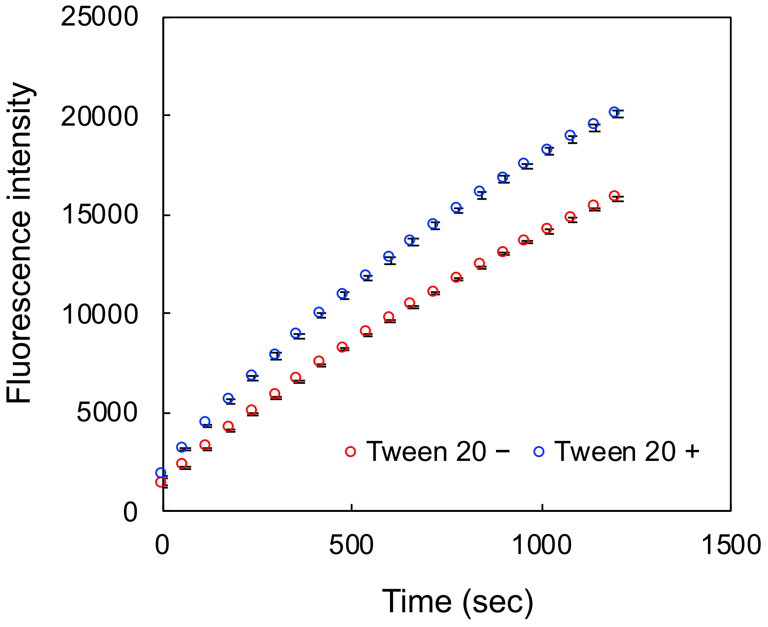

Figure S7 | Effect of the addition of Tween 20 on enzyme activity measurement of SmDPP7. Tyr-Tyr-MCA was used at 100 µM as a substrate in a reaction buffer consisting of 50 mM sodium phosphate buffer pH 7.0 and 5 mM EDTA. For Tween 20 +, 0.005% Tween20 was contained in the reaction buffer. Purified SmDPP7 was used in reaction buffer with a concentration of 2 nM. Enzyme reaction was conducted at 298 K for 20 min. The fluorescence intensity of the released MCA was measured with excitation at 355 nm and emission at 460 nm using an Infinite 200 PRO microplate reader. The standard deviations were obtained from three independent experiments.

Table S1 | Data collection statistics for SmDPP7.

Data set	Val-Tyr	Tyr-Tyr	Asn-Tyr	Phe-Tyr
Facility<br>Beamline	SPring-8<br>BL44XU	SPring-8<br>BL44XU	SPring-8<br>BL44XU	SPring-8<br>BL44XU
Wavelength (Å)	0.90000	0.90000	0.90000	0.90000
Detector	MX300HE	MX300HE	EIGER16M	EIGER16M
Crystal-to-detector distance (mm)	325.4	289.3	310.0	250.0
Rotation angle per image (°)	0.3	0.3	0.1	0.1
Total rotation range (°)	369.6	289.3	360	360
Exposure time per image (sec)	0.1	3<br>(gradual move mode)	0.1	0.1
Space group	$P2_1$	$P2_1$	$P2_1$	$P2_1$
Cell dimensions<br>*a* (Å)<br>*b* (Å)<br>*c* (Å)	 66.490<br>73.330<br>151.820	 67.850<br>74.320<br>153.320	 68.552<br>74.892<br>154.647	 68.035<br>74.555<br>153.869
α (°)<br>β (°)<br>γ (°)	90<br>95.16<br>90	90<br>94.15<br>90	90<br>94.50<br>90	90<br>94.44<br>90
Number of molecules per ASU	2	2	2	2
Mosaicity (°)	0.289	0.302	0.175	0.194
Resolution (Å)<br>(outer shell)	52.64-2.03<br>(2.07-2.03)	52.60-1.86<br>(1.89-1.86)	50.48-1.92<br>(1.95-1.92)	43.13-1.91<br>(1.94-1.91)
No. of observed reflections	700,660<br>(28,063)	531,519<br>(22,826)	823,378<br>(39,675)	816,227<br>(37,362)
No. of unique reflections	91,976<br>(3,919)	127,585<br>(6,238)	119,220<br>(5,629)	118,877<br>(5,580)
Completeness (%)	97.7 (83.9)	99.8 (98.0)	99.7 (94.8)	99.6 (93.9)
Redundancy	7.6 (7.2)	4.2 (3.7)	6.9 (7.0)	6.9 (6.7)
$I / \sigma_{(I)}$	8.5 (2.0)	13.0 (2.0)	7.4 (2.0)	7.0 (2.0)
CC_{half}	0.991 (0.597)	0.998 (0.637)	0.989 (0.632)	0.991 (0.631)
R_{merge} (I)	0.251 (1.179)	0.077 (0.698)	0.186 (1.199)	0.157 (1.012)
R_{meas} (I)	0.270 (1.271)	0.088 (0.820)	0.201 (1.295)	0.170 (1.098)
R_{pim} (I)	0.098 (0.470)	0.043 (0.421)	0.076 (0.485)	0.064 (0.421)
Wilson *B*-factor ($Å^2$)	17.4	19.9	14.5	25.2

Table S2 | Refinement statistics for SmDPP7.

Dataset	Val-Tyr	Tyr-Tyr	Asn-Tyr	Phe-Tyr
PDB ID	7DKB	7DKC	7DKD	7DKE
Resolution range (Å)	40.00-2.03	40.00-1.86	40.00-1.92	40.00-1.91
Completeness (%)	97.62	99.78	99.80	99.68
No. of reflections<br>working set<br>test set	 87,429<br>4,522	 121,344<br>6,217	 113,046<br>6,099	 112,986<br>5863
R-factor	0.201	0.171	0.202	0.207
Free *R*-factor	0.245	0.210	0.248	0.251
No. of protein atoms (avg. *B*-factors (Å^2))	10,658<br>(31.1)	10,660<br>(28.9)	10,669<br>(31.88)	10,667<br>(35.13)
No. of ligand atoms (avg. *B*-factors (Å^2))	40<br>(2 x 20)<br>(23.4)	50<br>(2 x 25)<br>(22.4)	42<br>(2 x 21)<br>(20.7)	48<br>(2 x 24)<br>(23.9)
No. of glycerol atoms (avg. *B*-factors (Å^2))	0	36<br>(6 x 6)<br>(44.7)	18<br>(6 x 3)<br>(30.4)	30<br>(6 x5)<br>(39.6)
No. of water molecules (avg. *B*-factors (Å^2))	461<br>(31.8)	1,361<br>(35.1)	1,162<br>(31.2)	861<br>(36.9)
Ramachandran plot statistics<br>favoured (%)<br>allowed (%)<br>outlier (%)	 1,340 (96.4)<br>48 (3.5)<br>2 (0.1)	 1,349 (97.1)<br>40 (2.9)<br>1 (0.1)	 1,325 (95.3)<br>61 (4.4)<br>4 (0.3)	 1,350 (97.1)<br>39 (2.8)<br>1 (0.1)
RMSD<br>bonds (Å)<br>angles (°)	 0.0097<br>1.58	 0.0105<br>1.61	 0.0090<br>1.55	 0.0097<br>1.57

Table S3 | Thermodynamic parameters of dipeptide binding to SmDPP7.

Ligand	H.I. of P2 a.a. [1,2]	K_d (μM)	ΔH (kcal/mol)	$-T\Delta S$ (kcal/mol)	ΔG (kcal/mol)
Asn-Tyr	-28	11.3 ± 0.2	-22.1 ± 0.5	15.3 ± 0.4	-6.70 ± 0.01
Tyr-Tyr	63	6.17 ± 0.39	-12.4 ± 0.3	5.32 ± 0.34	-7.11 ± 0.04
Leu-Tyr	97	3.01 ± 0.78	-12.5 ± 0.9	4.91 ± 1.02	-7.55 ± 0.15
Phe-Tyr	100	1.90 ± 0.23	-11.6 ± 0.4	3.79 ± 0.52	-7.81 ± 0.07
Val-Tyr*	76	> 40	-	-	-

The calorimetric data for the respective dipeptide bindings are shown in Figure S5. The diagram of thermodynamic parameters is represented in Figure 6. Standard deviations were obtained from three independent experiments. *The thermodynamic parameters were not determined due to low affinity (see Figure S5(e)). The hydrophobicity indexes (H.I.) of the amino acids were adopted from Sereda *et al.,* 1994[1] and Mohera *et al.,* 1995[2].

1. Sereda, T. J., Mant, C. T., Sönnichsen, F. D. & Hodges, R. S. Reversed-phase chromatography of synthetic amphipathic α-helical peptides as a model for ligand/receptor interactions Effect of changing hydrophobic environment on the relative hydrophilicity/hydrophobicity of amino acid side-chains. *J. Chromatogr. A* **676**, 139–153 (1994).

2. Monera, O. D., Sereda, T. J., Zhou, N. E., Kay, C. M. & Hodges, R. S. Relationship of sidechain hydrophobicity and α-helical propensity on the stability of the single-stranded amphipathic α-helix. *J. Pept. Sci.* **1**, 319–329 (1995).

Table S4 | Conservation of S2 subsite residues in S46 peptidases.

Residues of SmDPP7		Amino acid residue	Residue conservation (%)
S2 subsite	His83	His	100
	Gly205	Gly	89.6
		Ala	10.1
		Other	0.3
	Lys206	Lys	68.7
		Asn	11.5
		Other	19.8
	Phe207	Phe	78.9
		Tyr	20.6
		Other	0.5
	Gly208	Gly	99.9
		Other	0.1
	Gly209	Gly	70.9
		Ser	10.2
		Other	18.9
	Asp210	Asp	79.3
		Glu	19.8
		Other	0.9
	Arg218	Arg	98.8
		Other	1.2
	Thr220	Thr	79.2
		Other	20.8
	Asp222	Asp	100.0
	Phe671	Phe	99.3
		Other	0.7
S1 subsite	Asn332	Asn	49.6
		Lys	21.6
		Other	28.8
	Asp649	Asp	80.8
		His	18.8

		Other	0.3
	Ile650	Ile	56.3
		Thr	36.4
		Other	7.3
	Gly652	Gly	98.1
		Other	1.9
	Ala670	Ala	48.5
		Asn	32.3
		Other	19.2
	Gly673	Gly	60.8
		Arg	25.5
		Ser	12.4
		Other	1.4
	Ser677	Ser	43.3
		Ala	30.1
		Gly	24.7
		Other	1.8
	Val678	Leu	29.0
		Val	20.8
		Met	20.2
		Thr	16.5
		Ile	12.3
		Other	1.1
	Ser680	Gly	46.1
		Ser	36.3
		Other	17.6
	Asn681	Asp	76.9
		Other	23.1
	Met691	Thr	24.5
		Ala	21.3
		Asn	16.2
		Cys	15.6
		Ser	15.4

		Other	7.0
N-ter. recognition	Asn213	Asn	99.9
		Other	0.1
	Trp214	Trp	77.1
		Phe	18.8
		Other	4.1
	Asn328	Asn	99.5
		Other	0.5
	Asp672	Asp	99.8
		Other	0.2

Amino acid sequence variations were analysed for 4931 peptidases belonging to the S46 family. Amino acid sequence of PmDAP BII (UniProt accession numbers: V5YM14) was used as a query sequence. BLAST (Basic Local Alignment Search Tool) was used for searching the amino acid sequences of S46 peptidases. Multiple sequence alignment was conducted with the *Muscle* program and CLC genomics workbench 11 application. Residues located in the S2 subsite, S1 subsite, and N-terminus recognition of SmDPP7 are shown on the left. Dark green, green, and light green mean ≧80%, 80% > 60%, and ≦60% of conservation, respectively. "Other" represents the sum of the residues with conservation degree of under 10%.

Table S5 | Primers used for construction of mutant SmDPP7s.

Primer	Sequence 5' → 3'
K206A-Fw	GTCGGTgcgTTCGGCGGTGATGTGGAC
K206A-Rv	GCCGAAcgcACCGACGCTGCCCGGCGG
R218A-Fw	TGGCCGgcgCATACCGGTGATTTTTCT
R218A-Rv	GGTATGcgcCGGCCACATCCAGTTGTC
R218Q-Fw	TGGCCGcagCATACCGGTGATTTTTCT
R218Q-Rv	GGTATGctgCGGCCACATCCAGTTGTC
R218K-Fw	TGGCCGaaaCATACCGGTGATTTTTCT
R218K-Rv	GGTATGtttCGGCCACATCCAGTTGTC
T220A-Fw	CGTCATgcgGGTGATTTTTCTTTCTAT
T220A-Rv	ATCACCcgcATGACGCGGCCACATCCA
F671A-Fw	GGTCTGGCAgcgGATGGTAACTGGGAAAGT
F671A-Rv	ATCcgcTGCCAGACCAACCAGTTT

Underlined area represents overlap regions and lower-case letters represent mutation points.
